# Regulation of Protein Quality Control by UBE4B and LSD1 through p53-Mediated Transcription

**DOI:** 10.1371/journal.pbio.1002114

**Published:** 2015-04-02

**Authors:** Goran Periz, Jiayin Lu, Tao Zhang, Mark W. Kankel, Angela M. Jablonski, Robert Kalb, Alexander McCampbell, Jiou Wang

**Affiliations:** 1 Department of Biochemistry and Molecular Biology and Department of Neuroscience, Bloomberg School of Public Health and School of Medicine, Johns Hopkins University, Baltimore, Maryland, United States of America; 2 Biogen Idec, Cambridge, Massachusetts, United States of America; 3 Department of Neuroscience, Perelman School of Medicine, University of Pennsylvania, Philadelphia, Pennsylvania, United States of America; 4 Department of Neurology and Pediatrics, Perelman School of Medicine, University of Pennsylvania, Philadelphia, Pennsylvania, United States of America; University of Cambridge, UNITED KINGDOM

## Abstract

Protein quality control is essential for clearing misfolded and aggregated proteins from the cell, and its failure is associated with many neurodegenerative disorders. Here, we identify two genes, *ufd-2* and *spr-5*, that when inactivated, synergistically and robustly suppress neurotoxicity associated with misfolded proteins in *Caenorhabditis elegans*. Loss of human orthologs ubiquitination factor E4 B (UBE4B) and lysine-specific demethylase 1 (LSD1), respectively encoding a ubiquitin ligase and a lysine-specific demethylase, promotes the clearance of misfolded proteins in mammalian cells by activating both proteasomal and autophagic degradation machineries. An unbiased search in this pathway reveals a downstream effector as the transcription factor p53, a shared substrate of UBE4B and LSD1 that functions as a key regulator of protein quality control to protect against proteotoxicity. These studies identify a new protein quality control pathway via regulation of transcription factors and point to the augmentation of protein quality control as a wide-spectrum antiproteotoxicity strategy.

## Abstract 2

A new protein quality control regulatory pathway is identified in which a ubiquitin ligase and a lysine-specific demethylase act together on the transcription factor p53 to control protein degradation systems.

## Author Summary

To function properly, proteins must assume their correct three-dimensional shapes. There are numerous mechanisms within the cell, collectively referred to as protein quality control (PQC), that verify proper folding. If abnormal folding is detected, PQC can either help the protein to refold or target it for degradation. Failures in protein folding and PQC lead to the accumulation of misfolded proteins, which often self-associate into large aggregations that are thought to be the underlying cause of several neurodegenerative diseases. In this study, we use the roundworm *Caenorhabditis elegans* as a model to understand how cells handle disease-associated misfolded proteins. In a large-scale genetic screen, we discovered two suppressor genes, *ufd-2* and *spr-5*, which encode a ubiquitin ligase and a lysine-specific demethylase, respectively. When these two proteins are inactivated, we observe a marked reduction in the toxicity of several misfolded proteins. *ufd-2* and *spr-*5 are conserved in humans (UBE4B and LSD1, respectively), as are their effects on misfolded proteins. We show that UBE4B and LSD1 regulate the activity of protein degradation machineries including the proteasome and autophagosomes. Using microarrays and biochemical analyses, we identify p53 as a key downstream transcription factor that mediates the action of UBE4B and LSD1 on protein clearance. This work establishes p53 as a regulator of proteome integrity and uncovers a new protein quality control pathway that could potentially be exploited to increase the degradation of misfolded proteins in diseased cells.

## Introduction

Living organisms endure environmental stress and metabolic errors that inflict damage on macromolecules, including DNA and protein, which are either repaired or removed by quality control programs in the cell. Toxicity resulting from protein misfolding and aggregation, known as proteotoxicity, underlies many degenerative diseases, including those affecting the nervous system, such as Creutzfeldt-Jakob disease, Alzheimer disease, Parkinson disease, Huntington disease, frontotemporal dementia (FTD), and amyotrophic lateral sclerosis (ALS) [1,2]. To guard against proteotoxicity, the cell coordinates several major quality control systems, including molecular chaperones, the ubiquitin-proteasome system, and autophagy [3–6]. The regulation of these systems occurs on different scales, from individual proteins to the whole organism [7]. It is tantalizing to envisage enhancing the protein quality control systems to defend against proteotoxicity associated with neurodegenerative diseases. However, how the protein quality control might be harnessed in the cell to alleviate proteotoxicity-associated neurodegeneration is not yet fully understood.

Mutant Cu/Zn superoxide dismutase (SOD1), linked to ~20% of familial ALS, represents a simple molecular model for protein misfolding and aggregation. The wild-type (WT) SOD1 protein has a stable β-barrel structure with a two-state folding process, whereas mutant SOD1 proteins show a heightened propensity to aggregate in vitro and in vivo [8–10]. There is increasing evidence that heightened propensity to misfold and aggregate is a common feature of ALS/FTD-associated proteins, including TAR DNA binding protein 43 (TDP-43) and fused in sarcoma (FUS) [11–13]. Identifying mechanisms that suppress the toxicity of protein misfolding and aggregation may help elucidate the pathogenesis of neurodegenerative diseases and provide potential targets for correction.

Here we took advantage of a *Caenorhabditis elegans* model that expresses neuronal ALS-linked SOD1 mutant proteins and develops robust movement defects to perform an unbiased genetic screen for potent suppressors of the behavioral defects. We identified mutations in two genes, *ufd-2*, encoding a ubiquitin ligase, and *spr-5*, encoding a lysine-specific demethylase, that synergistically attenuate the neurotoxicity of mutant human SOD1 proteins. The actions of the suppressor genes are conserved in *Drosophila*, and they protect against proteotoxicity initiated by diverse mutant proteins, including TDP-43, FUS, and the polyglutamine (polyQ) tract. Furthermore, we found human orthologs of these modifiers to be part of a pathway regulating protein quality control in mammalian cells. Further analysis showed that this pathway acts through the transcription factor p53, which mediates cellular stress responses. Together, these results identify a new mechanism involving previously unrecognized players, which the cell utilizes to augment protein quality control.

## Results

### Isolation of Suppressors of Mutant SOD1-Induced Neurotoxicity in *C*. *elegans*

**Fig 1:**
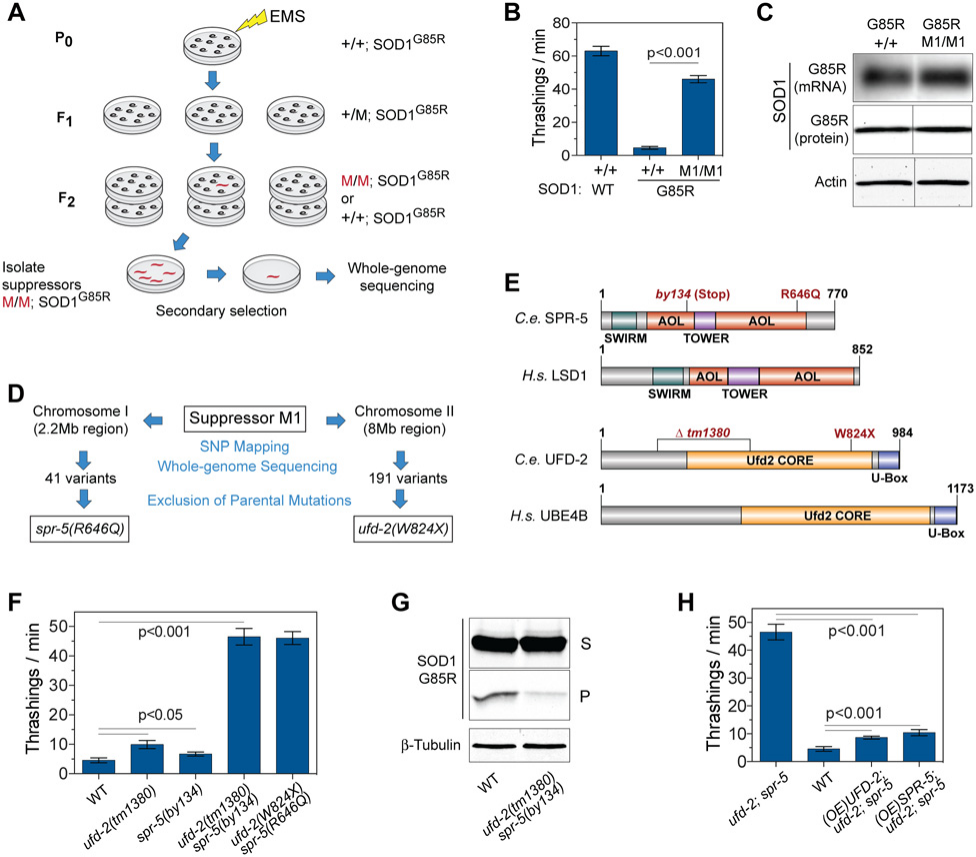
Identification and characterization of a robust suppressor that ameliorates the locomotion defects in the *C*. *elegans* model of SOD1-associated ALS. (**A**) Workflow of the suppressor screen identifying mutant *C*. *elegans* (red) with saliently improved movement. (**B**) Locomotor behavior, measured by thrashing rates in liquid medium, in the *C*. *elegans* strains with neuronal expression of human WT SOD1 or ALS-linked mutant SOD1^G85R^, in the presence (M1/M1) or absence (+/+) of the suppressor mutation (*n* = 16). (**C**) Northern (top panel) and western (middle, bottom panels) blot analyses of total RNA and protein from SOD1^G85R^ strains, with (M1/M1) or without (+/+) suppressor mutations, demonstrating that the levels of SOD1^G85R^ mRNA and total protein are not changed by the suppressor mutation (M1/M1). The western blot lanes are from the same gel and exposure. (**D**) Sequence analysis of the M1 strain, revealing that independent mutations in two genes, a lysine-specific demethylase, *spr-5 (R646Q)*, and a ubiquitin ligase, *ufd-2 (W824X)*, are required for the full suppressor phenotype. (**E**) *C*. *elegans* SPR-5 and UFD-2 and their mammalian homologs lysine-specific demethylase 1 (LSD1) and ubiquitination factor E4 B (UBE4B) share all major protein domains. These include the Swi3-Rsc8-Moira (SWIRM), amine oxidase-like (AOL), and TOWER domains in LSD1/SPR-5 and the Ufd2 Core and U-box domains in UBE4B/UFD-2. Positions of the missense, nonsense, and deletion mutations in mutant *C*. *elegans* are indicated. (**F**) Locomotor behavior, measured by thrashing rates, of the *C*. *elegans* carrying the SOD1^G85R^ transgene on the normal background (WT), with the null mutation of either *ufd-2(tm1380)* or *spr-5(by134)*, the double mutation *ufd-2(tm1380);spr-5(by134)*, or the M1 suppressor *ufd-2(W824X);spr-5(R646Q*) (*n* = 16). (**G**) Western blotting of the supernatant (S) and pellet (P) protein fractions from the *C*. *elegans* carrying the SOD1^G85R^ transgene with the double mutation *ufd-2(tm1380);spr-5(by134)* compared with controls. While SOD1^G85R^ protein levels are unchanged in the S fraction, those in the P fraction are decreased in the double suppressor mutant. The P fraction represents only about 1.7% of total SOD1^G85R^ in the WT sample; therefore, a higher ratio of the P fraction relative to the S fraction (approximately 2:1) is used for the western analysis. (**H**) The overexpression (OE) of WT UFD-2 or SPR-5 in the *C*. *elegans* nervous system blocks the protection conferred by the double mutation *ufd-2(tm1380);spr-5(by134)* (*n* = 16). Data represent means ± SEM. The numerical data used to make this figure can be found in S1 Data.

To better understand the regulatory mechanisms that mitigate an increased load of misfolded proteins, we conducted a forward genetic screen for cellular factors that alleviate such stress and relieve cells from proteotoxic insults. This screen took advantage of a *C*. *elegans* model of ALS, in which the neuron-directed expression of the ALS-linked, G85R mutant human SOD1 (SOD1^G85R^) protein leads to its aggregation into misfolded soluble oligomers and larger insoluble aggregates [14,15]. Misfolded SOD1^G85R^ protein is highly toxic, leading to age-dependent synaptic dysfunction, neurodegeneration, and severely impaired movement in the worms [14]. This severe locomotor defect allowed us to perform a large-scale screen for genes that suppress neurodegeneration and improve worm locomotion. In these experiments, we treated homozygous transgenic SOD1^G85R^ *C*. *elegans* with ethyl methanesulfonate (EMS) to induce genomic mutations, and the mutagenized P_0_ hermaphrodites were allowed to self-reproduce for two generations (Fig. 1A). Next, in the F_2_ offspring, which contain both heterozygous and homozygous suppressor mutations, we selected individual *C*. *elegans* based on a salient improvement in the locomotion on a background of poorly moving populations. The potential suppressor clones were bred through until 100% of progeny showed phenotypic improvements and were then subjected to further analysis (Fig. 1A).

After screening >10^5^ haploid genomes, we isolated hundreds of independent strains with markedly improved locomotion. Most of these strains were dismissed upon closer examination because they showed a reduction in the expression of a green fluorescent protein (GFP) reporter gene that had been coinjected as an internal reference and expressed independently in the pharynx, suggesting silencing of the transgene cassette. Among the few remaining suppressor strains that survived this test, one designated M1 showed potent suppression of the locomotion defect when compared with the parental SOD1^G85R^ line, reaching ~76% of the locomotion robustness of the SOD1-WT transgenic line (Fig. 1B and S1 Movie). Such strong recovery of locomotion was apparently not a consequence of diminished SOD1^G85R^ transgene expression because SOD1^G85R^ mRNA and protein levels were unchanged between the parental and M1 mutant strains (Fig. 1C). Further segregation analysis of M1 indicated that more than one genetic locus, in addition to the SOD1 transgene on chromosome IV, was linked to the suppressor phenotype, suggesting a rare multigenic suppressor underlying the suppressor phenotype.

To map and identify genes responsible for the suppression of the locomotor defect, we carried out single-nucleotide polymorphism (SNP) mapping [16]. SNP mapping localized the M1 suppressor mutations to two linkage regions: a 2.2-Mb interval on chromosome I and an 8-Mb interval on chromosome II (Fig. 1D). Next, we performed two rounds of deep sequencing on the M1 strain genomic DNA [17], attaining a 27-fold coverage. When the M1 genomic DNA sequencing data was aligned with the *C*. *elegans* reference genome, we found over 200 variants in the two linkage regions. Next, we performed deep sequencing of the parental strain carrying only the SOD1^G85R^ transgene, with 7.5-fold coverage. Comparison of the parental and M1 genomic sequences indicated that most of the nonreference variants existed prior to the EMS mutagenesis and thus were not responsible for the suppressor phenotype. Our analysis pinpointed two variants as likely candidates for the suppressor mutations in M1: in the chromosome I linkage region, there is only one missense mutation, G1937A, resulting in a single amino acid change (R646Q) in the gene *suppressor of presenilin 5* (*spr-5*); and on chromosome II, among the few remaining variants is one nonsense mutation, G2472A, which results in a premature stop (W824X) in the gene *ubiquitin fusion degradation 2* (*ufd-2*) (Fig. 1D and 1E).

To examine the role of the double mutations *ufd-2(W824X)* and *spr-5(R646Q)* in the suppression of mutant SOD1-mediated neurotoxicity, we performed a series of genetic, biochemical, and behavioral analyses. *ufd-2* encodes a U-box type ubiquitin ligase, and the W824X mutation results in a truncated protein lacking the C-terminal U-box (Fig. 1E). *spr-5* encodes a lysine-specific demethylase, and the R646Q substitution occurs at a highly conserved residue in the C-terminal portion of an amine oxidase-like (AOL) domain (Fig. 1E). While either *ufd-2(W824X)* or *spr-5(R646Q)* alone did not lead to the strong locomotor defect-suppressing phenotype in the M1 strain, the double mutation *ufd-2(W824X)* and *spr-5(R646Q)* segregated perfectly with the M1 phenotype, recapitulating the full rescuing effect of the suppressor.

To confirm *ufd-2* and *spr-5* as the suppressor genes, we obtained independent null alleles of the two genes: a deletion mutation, *ufd-2(tm1380)*, that lacks 80% of the protein at the C-terminus [18] and a nonsense mutation, *spr-5(by134)*, that lacks the C-terminal half of the protein (Fig. 1E) [19]. When crossed to the mutant SOD1 strain, the single allele of *ufd-2(tm1380)* provided a moderate, 2-fold locomotor improvement, and less improvement was seen for the single allele of *spr-5(by134)* (Fig. 1F). However, combining the alleles of *spr-5(by134)* and *ufd-2(tm1380)* completely recapitulated the strong locomotor-defect-suppressing phenotype observed in the M1 strain (Fig. 1F). Total levels of SOD1^G85R^ protein were similar among the WT, single-, and double-mutant strains (S1B Fig.). However, further analysis after fractionation by solubility revealed that the insoluble level of SOD1^G85R^, which accounts for less than 2% of total proteins, was decreased by the *spr-5(by134);ufd-2(tm1380)* mutations, while the soluble level of SOD1^G85R^ remained unchanged (Fig. 1G). Finally, we found that restoring the function of either *ufd-2* or *spr-5* alone by expressing transgenic wild-type *ufd-2* or *spr-5* under a neuron-specific promoter from the synaptobrevin (*snb-1*) gene completely blocked the protection in the M1 strain (Fig. 1H), indicating that it was the loss of function in these two genes and not any other background mutation that was responsible for the suppressor phenotype. Taken together, these results establish that the synergistic loss of *ufd-2* and *spr-5* creates a potent novel suppressor of the neurodegenerative phenotypes in the SOD1 *C*. *elegans* model of ALS, which we have termed the *s* *pr-5*– and *u* *fd-2*–dependent neurodegeneration suppressor (SUNS).

### Loss of *ufd-2* and *spr-5* Suppresses Neurotoxicity of Diverse Proteotoxic Stressors in Invertebrate Models

**Fig 2:**
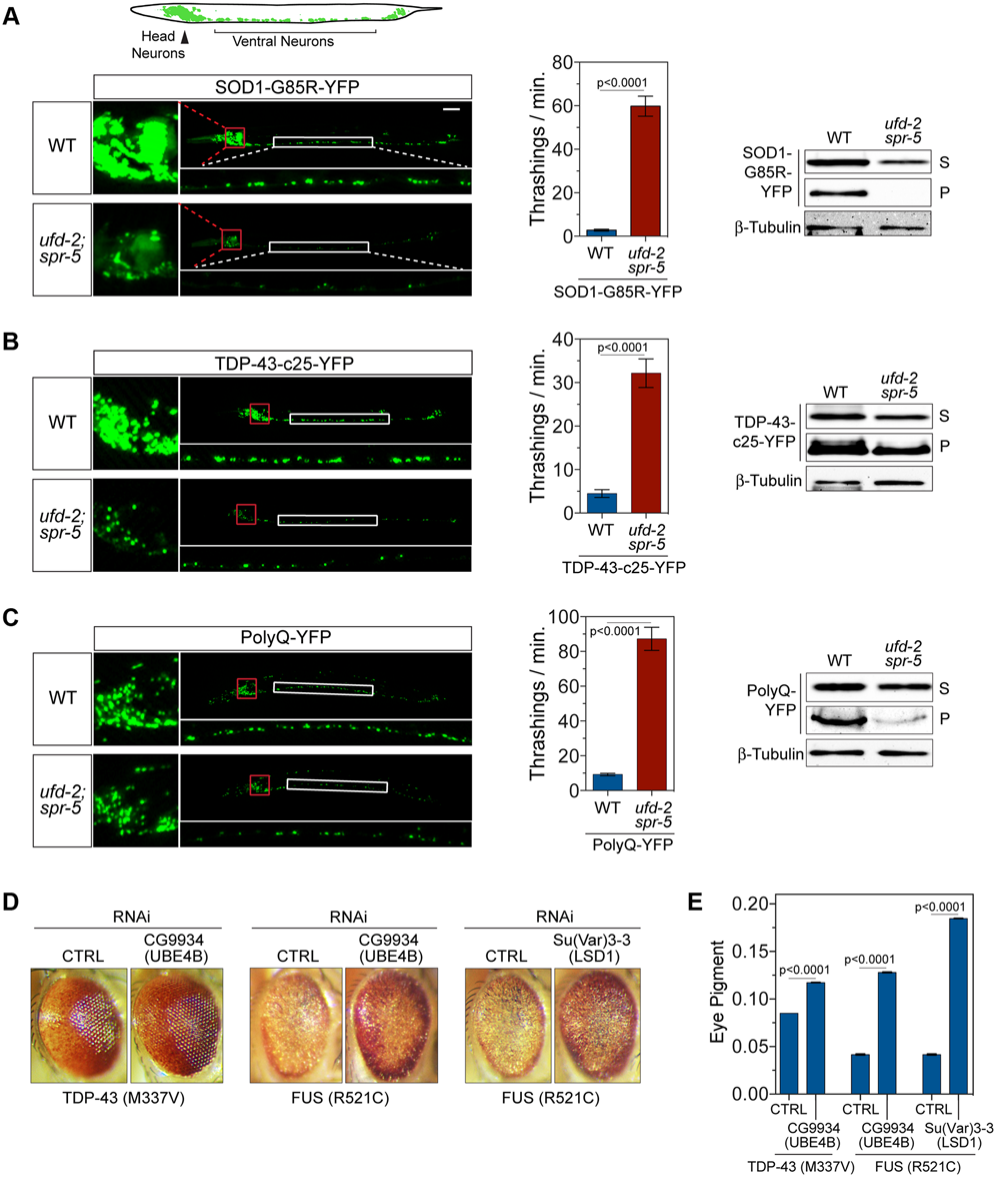
Suppression of neurodegeneration associated with diverse misfolded proteins in invertebrate models by *ufd-2* and *spr-5* loss-of-function mutations. (**A**) Top: schematic drawing depicts pan-neuronal expression of YFP in head and ventral neurons in the context of the *C*. *elegans* body plan. Left: micrographs show the SOD1^G85R^-YFP proteins expressed in WT or the *spr-5(by134);ufd-2(tm1380)* mutant background. The double-mutant worms show a marked decrease in protein aggregation in neurons. Enlarged sections of head neurons (red framed) and ventral cord neurons (white framed) are shown. Middle: quantification of locomotion in the *spr-5(by134);ufd-2(tm1380)* and the WT *C*. *elegans* with neuronal expression of SOD1^G85R^-YFP (*n* = 30). Right: a decrease in the protein levels of SOD1^G85R^-YFP in the presence of *spr-5(by134);ufd-2(tm1380)* is shown by western blots of the supernatant (S) and the pellet (P) fractions. (**B and C**) Analyses of the *spr-5(by134);ufd-2(tm1380)* and the WT *C*. *elegans* with neuronal expression of TDP-43-c25-YFP (*n* = 30) or PolyQ-YFP (*n* = 12) as in (A). (**D**) Neurodegenerative rough-eye phenotype in adults is alleviated by the knockdown of the *Drosophila* homologs of *ufd-2*/UBE4B and *spr-5*/LSD1—CG9934 and Su(Var)3-3, respectively, compared to the control (CTRL). Eye-specific expression of TDP-43^M337V^, FUS^R521C^, and RNA interference (RNAi) was driven by GMR-Gal4. (**E**) *Drosophila* eye pigment quantitation. Data represent means ± SEM. The numerical data used to make this figure can be found in S1 Data.

Next, we asked whether the SUNS suppressor genes affect the aggregation and toxicity of other misfolded proteins. In transparent *C*. *elegans* models, yellow fluorescent protein (YFP) fusions of several disease-relevant, aggregation-prone proteins, such as SOD1^G85R^-YFP [14], TDP-43^c25^-YFP [20], and PolyQ-YFP [21], have been used to facilitate the visualization of their protein aggregation. These fusion proteins form fluorescent protein aggregates readily visible in live *C*. *elegans*, and, when present in neurons, these protein aggregates correlate with the toxicity to the animals as manifested in their locomotor defects [14,20,21]. To determine whether the SUNS mutant reduces the toxicity associated with these protein aggregates, we introduced the double-null mutations *ufd-2(tm1380);spr-5(by134)* into the strains that pan-neuronally express SOD1^G85R^-YFP, TDP-43^c25^-YFP, or PolyQ-YFP. Indeed, loss of *ufd-2* and *spr-5* function resulted in a marked reduction in the neuronal protein aggregation when compared with controls. Reduction in the number and intensity of protein aggregates was evident in the change in the fluorescent inclusions in the head and ventral cord regions of the SUNS-mutant *C*. *elegans* (Fig. 2A–2C). Consistently, the locomotor phenotypes in these *C*. *elegans* strains were significantly improved by the introduction of the SUNS mutations, *ufd-2(tm1380);spr-5(by134)* (Fig. 2A–2C).

To investigate the biochemical states of the misfolded proteins in the *C*. *elegans* models, we performed a protein solubility assay by differentially extracting and sedimenting worm lysates into soluble supernatants and insoluble pellets. The worm pellet fraction is enriched with sedimentable large SOD1 protein aggregates, whereas the supernatant fraction contains smaller aggregates and oligomeric species (S1A Fig.) [14,15]. Western blot analysis of both supernatant and pellet fractions displayed a significant decrease in the levels of misfolded SOD1^G85R^-YFP, TDP-43^c25^-YFP, and PolyQ-YFP in the *ufd-2(tm1380);spr-5(by134)* double mutant when compared with controls (Fig. 2A–2C). Compared with the untagged SOD1^G85R^ (the soluble level of which was unchanged, S1B Fig.), SOD1^G85R^-YFP was significantly reduced in its soluble fraction by *ufd-2(tm1380);spr-5(by134)*. This suggests that soluble SOD1^G85R^-YFP proteins were degraded more rapidly than the untagged SOD1^G85R^, consistent with the notion that the YFP tag may increase the misfolding of fusion proteins and therefore decrease their total protein levels (S1C Fig.). Taken together, these data indicate that the *ufd-2;spr-5* double mutations are capable of reducing the proteotoxicity of various misfolded proteins associated with neurodegeneration, suggesting a wide-spectrum effect of the suppressor.

To investigate whether this effect was evolutionarily conserved, we assessed the actions of *ufd-2* and *spr-5* in transgenic *Drosophila* models expressing known ALS disease-causing human TDP-43^M337V^ or FUS^R521C^ proteins. These models have been previously shown to cause photoreceptor degeneration and rough-eye phenotypes [22,23]. We found that knockdown of *CG9934*, a *Drosophila* homolog of *C*. *elegans ufd-2* and human ubiquitination factor E4 B (UBE4B), rescues the TDP-43^M337V^-induced degenerative rough-eye phenotype, resulting in a smoother eye appearance (Fig. 2D, left) and restoration of pigmentation (Fig. 2E). Similarly, knockdown of the *ufd-2* homolog *CG9934* corrected the ommatidial defects (Fig. 2D, middle) and pigmentation loss induced by FUS^R521C^ (Fig. 2E). Additionally, we observed that knockdown of *Drosophila* Su(Var)3-3, the homolog of *C*. *elegans spr-5* and human lysine-specific demethylase 1 (LSD1), rescued the degenerating eye phenotypes induced by FUS^R521C^ (Fig. 2D, right, and Fig. 2E). Neither of the suppressors changed the protein expression levels of TDP-43^M337V^ or FUS^R521C^ (S1D Fig.). Taken together, these results indicate that the loss of *ufd-2* and *spr-5* homologs also suppresses proteotoxicity-related phenotypes in diverse *Drosophila* models.

### Knockdown of Mammalian UBE4B and LSD1 Enhances Clearance of Misfolded Proteins

**Fig 3:**
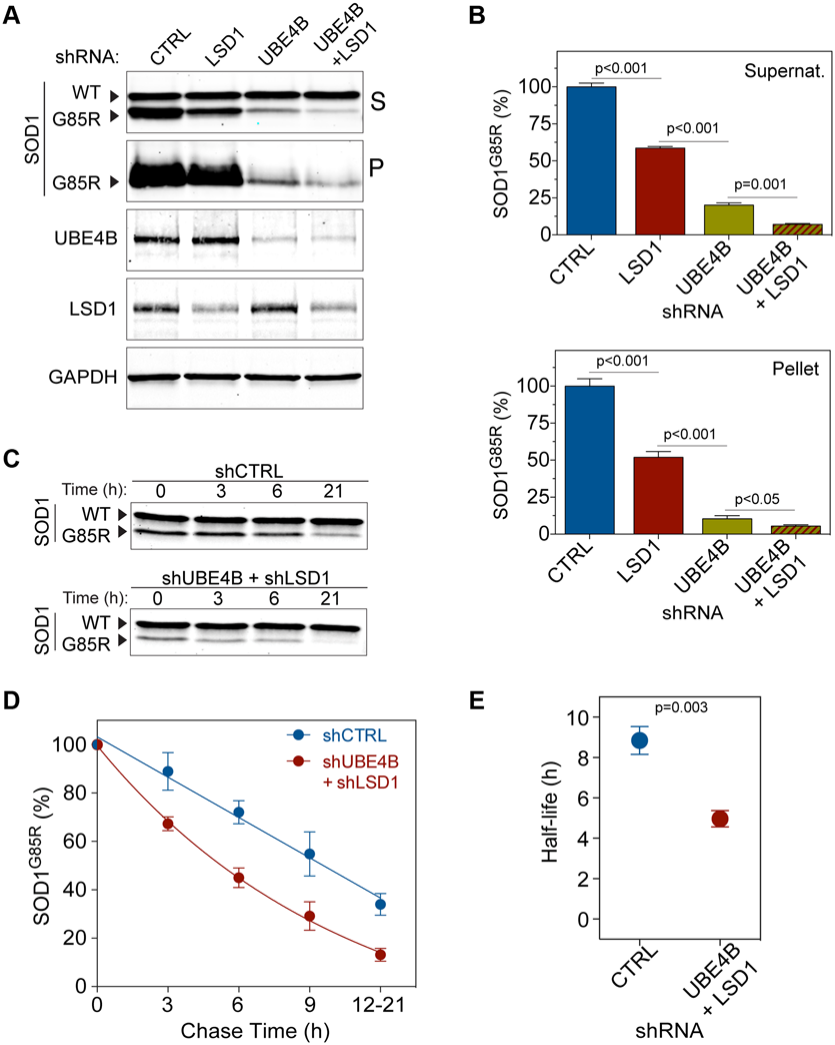
UBE4B and LSD1 double-knockdown accelerates SOD1^G85R^ protein degradation. (**A**) Western blots of cell lysates derived from mock (CTRL), single UBE4B or LSD1, or double UBE4B and LSD1 knockdowns. Supernatant (S) and pellet (P) fractions were probed with indicated antibodies. While the LSD1 or UBE4B single-knockdown reduces the SOD1^G85R^ aggregates in both supernatant and pellet fractions, the combined knockdown produces the strongest reduction in the aggregates. (**B**) Quantification of SOD1^G85R^ protein levels by western blotting (A). *n* = 3 (supernatant); *n* = 8 (pellet). (**C**) Western blots of a representative cycloheximide chase experiment to determine SOD1 protein half-lives in the double UBE4B and LSD1 knockdown cells versus controls. (**D**) Quantification of SOD1^G85R^ clearance, as analyzed by western blotting in (C). The graph indicates the relative band intensity of SOD1^G85R^ at each chase time point. *n* = 5; Overall *p* = 0.02 (paired *t* test, CTRL versus UBE4B and LSD1 double knockdown). Individual *p* = 0.03 (3 h), *p* = 0.003 (6 h), *p* = 0.06 (9 h), and *p* = 0.004 (12–21 h). (**E**) The half-life of SOD1^G85R^ is reduced from 8.5 h to 5 h upon knockdown of UBE4B and LSD1. Data represent means ± SEM. The numerical data used to make this figure can be found in S1 Data.

Homologs of *ufd-2* and *spr-5* are present in all eukaryotes. UBE4B and LSD1 are the human orthologs of *ufd-2* and *spr-5*, respectively. UBE4B and LSD1 share 32% and 29% sequence identity with *ufd-2* and *spr-5*, respectively, and all the major protein domains are conserved (Fig. 1E). To determine whether UBE4B and LSD1 affect protein aggregation in mammals, we used a protein solubility assay that was established to characterize the aggregation of mutant SOD1 in HEK293T cells (S2A Fig.) [9,24]. This assay utilizes the aggregation-prone SOD1^G85R^ protein, which migrates faster on SDS-PAGE than its WT counterpart, as a reporter of protein aggregation, with WT SOD1 serving as an internal control. We knocked down UBE4B and/or LSD1 with multiple RNA interference (RNAi) oligonucleotides and analyzed the levels and solubility of SOD1^G85R^ protein in HEK293T cells. Cell lysates were subjected to ultracentrifugation to separate insoluble pellets, which contain large and sedimentable SOD1^G85R^ aggregates, from soluble supernatants, which contain correctly folded native proteins, misfolded proteins, and small oligomeric aggregates (S1A Fig.). The WT SOD1 protein remained in the supernatant (S) in all tested conditions, but a significant portion of SOD1^G85R^ protein (25%–30%) was enriched in the insoluble pellet (P) fraction (Fig. 3A). Knockdown of both UBE4B and LSD1 significantly decreased levels of SOD1^G85R^ in both supernatant and pellet fractions (Fig. 3B), consistent with reduction of total SOD1^G85R^ proteins (S2B Fig. and S2C Fig.). The UBE4B/LSD1 knockdown did not affect the protein levels of WT SOD1. These results were consistent with the notion that the mutant SOD1^G85R^ had a much larger fraction of misfolded and aggregated proteins that were sensitive to UBE4B- and LSD1-dependent clearance than the WT SOD1 protein. In agreement with the observations in *C*. *elegans* and *Drosophila*, the effects of UBE4B/LSD1 knockdown on enhanced protein clearance was not specific to mutant SOD1^G85R^ but also occurred with other aggregation-prone proteins, including TDP-43^Q331K^ (S2D Fig. and S2E Fig.), indicative of a general effect on protein quality control. Additionally, single knockdown of either LSD1 or UBE4B also resulted in decreased steady-state levels of SOD1^G85R^, with the UBE4B knockdown producing a stronger effect than LSD1 (Fig. 3B). However, the double knockdown produced an even more pronounced decrease in the SOD1^G85R^ levels, indicating an additive effect between UBE4B and LSD1.

To determine whether this decrease in the levels of misfolded and aggregated proteins was a consequence of increased degradation of proteins, we performed cycloheximide chase experiments. Cells were transfected with SOD1^G85R^ together with either nontargeting control small hairpin RNAs (shRNAs) or a mix of UBE4B and LSD1 shRNAs, and the clearance of the SOD1^G85R^ protein was quantified. Cycloheximide was used to block de novo translation, and the amount of SOD1^G85R^ protein remaining in the supernatant at the indicated time points after the translation block was determined by SDS-PAGE and western blotting (Fig. 3C). The UBE4B and LSD1 double knockdown decreased the half-life of SOD1^G85R^ from 8.5 h to 5 h, indicating that increased clearance of the mutant protein underlies the reduction of the protein aggregates (Fig. 3D and 3E). Similarly, the reduction of TDP-43^Q331K^ by knockdown of UBE4B and LSD1 was also attributable to increased protein clearance, as shown by cycloheximide chase experiments (S2F Fig. and S2G Fig.), further suggesting that a general enhancement of protein quality control is the consequence of the loss of function of the suppressor genes.

### Knockdown of UBE4B and LSD1 Activates p53-Mediated Transcription

To identify the downstream effectors of UBE4B and LSD1 in the antiproteotoxicity pathway, we performed a comprehensive transcriptional analysis using the cell-based SOD1 misfolding model. We treated HEK293T cells with shRNAs targeting either UBE4B or LSD1 alone or UBE4B and LSD1 simultaneously in the presence of SOD1^G85R^ proteins. Upon confirmation of reduction in UBE4B and LSD1 protein levels, total RNA was isolated and subjected to microarray profiling of the whole human transcriptome (S3A Fig.). In triplicate samples of the three knockdown conditions and nontargeting controls, differentially regulated genes and pathways were analyzed in unbiased approaches to identify those that convey the UBE4B/LSD1-mediated activation of protein quality control systems.

**Fig 4:**
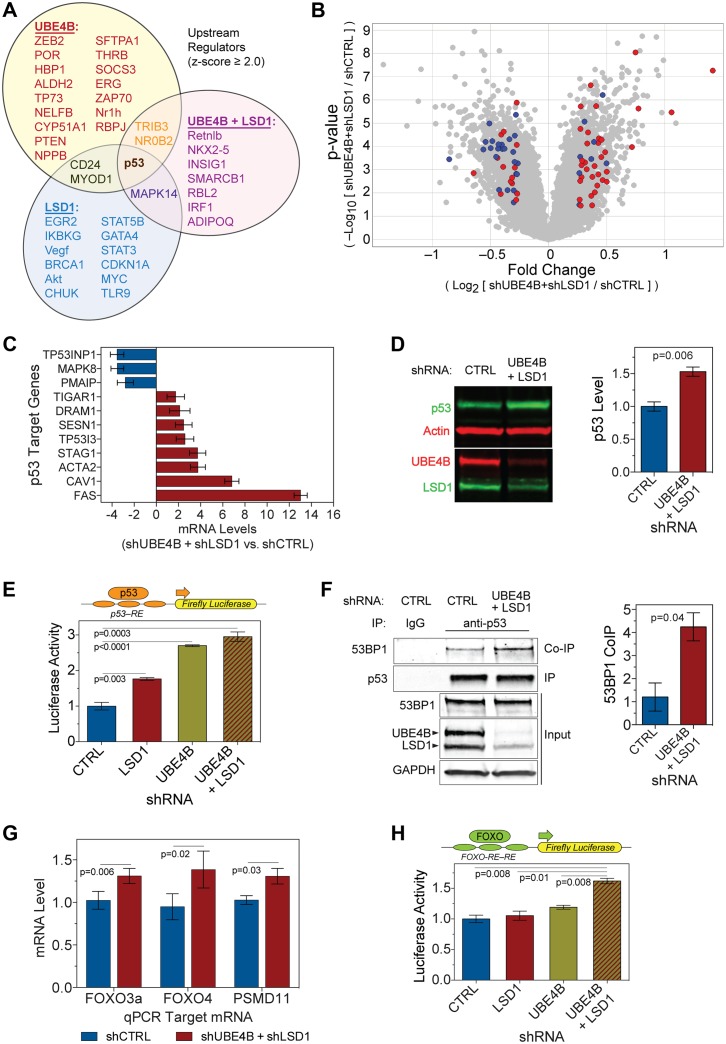
UBE4B and LSD1 knockdown activates transcription mediated by p53. (**A**) Venn diagram of upstream activators (z-score ≥ 2) that are differentially activated in single—UBE4B or LSD1—knockdowns and double UBE4B and LSD1 knockdowns, compared with the control. Activation state of an upstream regulator is predicted from differential mRNA levels of its downstream target genes. (**B**) The volcano scatter plot indicates fold changes in the levels of gene transcripts affected differentially by the UBE4B and LSD1 double knockdown versus control shRNA. Gray spots represent 22,148 annotated transcripts. Red spots are predicted p53-activated targets, and blue spots are predicted p53-inhibited targets. The enrichment of red spots in the up-regulated genes (right upper quadrant, >1.2-fold change, *p* ≤ 0.04) and blue spots in the down-regulated genes (left upper quadrant) indicates that the p53-mediated transcription is activated by the UBE4B and LSD1 double knockdown. (**C**) RT-qPCR validation of the expression levels of representative p53 target genes in the UBE4B and LSD1 double-knockdown samples. *n* = (2 to 6), *p* ≤ 0.04. (**D**) The p53 protein level is significantly increased by UBE4B and LSD1 double knockdown. Left: representative western blots showing p53 levels, the knockdown of UBE4B and LSD1, and the actin control in HEK293T cells. Right: quantification of the p53 protein levels normalized against actin (*n* = 3). (**E**) The p53-mediated transcriptional activity is measured by a luciferase reporter under the control of a p53-response element promoter, which was transfected into HEK293T cells 72–96 h after the initiation of the UBE4B and LSD1 single or double knockdowns (*n* = 6). (**F**) Increased interaction of p53 with its activating partner 53BP1 in response to UBE4B and LSD1 knockdown. Left: representative western blots of 53BP1 co-immunoprecipitation. Right: quantification of the 53BP1 co-immunoprecipitation (*n* = 2). Data represent means ± SEM. (**G**) RT-qPCR validation of the expression levels of FOXO3a, FOXO4, and PSMD11 (*n* = 6). (**H**) The FOXO3a-mediated transcriptional activity was measured with a luciferase reporter under the control of a FOXO-response element promoter. HEK293T cells were transfected with shRNAs for control, LSD1, UBE4B, or both LSD1 and UBE4B, followed by transfection of the luciferase reporter together with a constitutively active form of FOXO3a (TM) 72–96 h later (*n* = 6). Data represent means ± SEM. The numerical data used to make this figure can be found in S1 Data.

The most intriguing observation in our unbiased microarray analysis did not concern individually regulated genes but instead related to the upstream regulators that elicited characteristic pattern of mRNA level changes in a whole pathway or network. By employing the Ingenuity Pathway Analysis (IPA) algorithm to compare the predicted pattern of changes and the actual changes in these genes in our microarray profiles, we identified a number of upstream regulators whose downstream targets are significantly changed (z-score ≥ 2.0) in UBE4B and LSD1 single or double knockdowns (Fig. 4A). Among these upstream regulators, only a few were shared by more than one experimental condition, and remarkably, p53 was the only upstream regulator common to all three conditions (Fig. 4A and S1 Table). In the UBE4B and LSD1 double-knockdown condition, a large number of p53 target genes were affected, and importantly, a large fraction were changed in the directions that statistically suggest an activation of the p53 transcription factor (Fig. 4B, S3B Fig., S3C Fig., S2 Table). We examined a sample of 11 p53 target genes in HEK293T cells and confirmed by reverse transcription-quantitative polymerase chain reaction (RT-qPCR) that their expression levels were consistent with the microarray dataset (Fig. 4C). Since UBE4B is a ubiquitin ligase that decreases the stability of p53 [25–27], we examined the total protein levels of p53 in the UBE4B and LSD1 double-knockdown cells. We detected significantly increased p53 protein levels in the UBE4B and LSD1 double-knockdown cells when compared with the mock-knockdown control, indicating a stabilization of the p53 protein (Fig. 4D).

To further confirm that the single or double knockdowns of UBE4B and LSD1 were activating p53-mediated transcription, we used a firefly luciferase (p53RE-luc) reporter construct carrying p53-responsive elements in its promoter. Knockdown of either UBE4B or LSD1 increased p53 transcriptional activity. However, the simultaneous knockdown of both UBE4B and LSD1 resulted in an even higher p53 transcriptional activity (Fig. 4E), consistent with the synergistic antiproteotoxicity effects of knocking down both UBE4B and LSD1. To examine if the increased luciferase activity reflected p53-dependent transcriptional activation, we expressed MDM2, an E3 ubiquitin ligase and negative regulator of p53, or β-galactosidase as a control, together with the p53 activity reporter. The introduction of MDM2 significantly reduced p53-dependent transcriptional activation of the luciferase reporter under the UBE4B and LSD1 double-knockdown condition (S4A Fig.), confirming the specificity of the regulation of p53 by UBE4B and LSD1.

LSD1 demethylates the p53 protein, and loss of LSD1 increases K370-p53 dimethylation, an activating post-translational modification specifically recognized by 53BP1 [28]. To determine whether the activation of p53 resulted partially from its enhanced interaction with 53BP1, we co-immunoprecipitated p53 and 53BP1 from HEK293T cells in which LSD1 and UBE4B were previously knocked down. An increased amount of 53BP1 was pulled down by an equal amount of p53 protein in the double-knockdown cells when compared with the control, indicating an increased interaction between p53 and its coactivator, 53BP1 (Fig. 4F). To confirm directly that the dimethylation on the p53 protein is increased, we performed western blotting using a dimethyl K370-p53-specific antibody, and detected an increase in the level of dimethylated p53 in the double-knockdown samples compared with the control and compared with the total p53 (S4B Fig.). In sum, these data demonstrate that p53, as a transcription factor, is significantly elevated and activated by the knockdown of UBE4B and LSD1.

Among the genes that were up-regulated by the UBE4B and LSD1 double knockdown in our microarray data set, there were a few that had been reported to be important for protein quality control, including forkhead box O3 (FOXO3a), FOXO4, and proteasome 26S subunit non-ATPase11 (PSMD11) (S3C Fig. and S2 Table). FOXOs are a family of transcription factors invoked in protein quality control [20,29,30], and the 19S proteasome component PSMD11 is a critical regulator of proteasome activity [31,32]. It is notable that FOXO3a is transcriptionally up-regulated by p53 [33], and PSMD11 is transcriptionally induced by FOXOs [31,32]. We confirmed through RT-qPCR that FOXO3a, FOXO4, and PSMD11 were all transcriptionally up-regulated when UBE4B and LSD1 were knocked down (Fig. 4G), linking these positive regulators of protein quality control downstream of p53 to the UBE4B- and LSD1-dependent antiproteotoxicity activity.

Since FOXO3a is a transcription factor downstream of p53 and positively regulates protein quality control, we examined the FOXO3a-mediated transcriptional activity by employing a previously established luciferase reporter that is driven by the forkhead-responsive element (FHRE) [34]. A constitutively active form of FOXO3a, FOXO3a-TM, was co-expressed with the FHRE-luciferase reporter in HEK293T cells to measure the transcriptional activity of this particular FOXO member. Consistent with the p53 activation, we found that the FOXO3a activity is induced most strongly when both UBE4B and LSD1 are simultaneously knocked down (Fig. 4H). Similar results were observed with another FOXO-family member, FOXO1 (S4C Fig.). The *daf-16* gene is the sole *C*. *elegans* ortholog of the mammalian FOXO family [35,36]. To examine a role of *daf-16*/FOXO in the SUNS pathway in *C*. *elegans*, we built a quadruple mutant strain carrying the SOD1^G85R^ transgene, the SUNS mutations *spr-5(by134)* and *ufd-2(tm1380)*, and loss-of-function mutation *daf-16(mu86)*. In *C*. *elegans* locomotor assays, *daf-16(mu86)* partially blocked the rescuing effect of the SUNS mutations on SOD1^G85R^ toxicity, suggesting that the *daf-16* gene activity is partially required for the suppressor activity (S4D Fig.).

### Knockdown of UBE4B and LSD1 Activates Proteasomal Degradation and Autophagy

**Fig 5:**
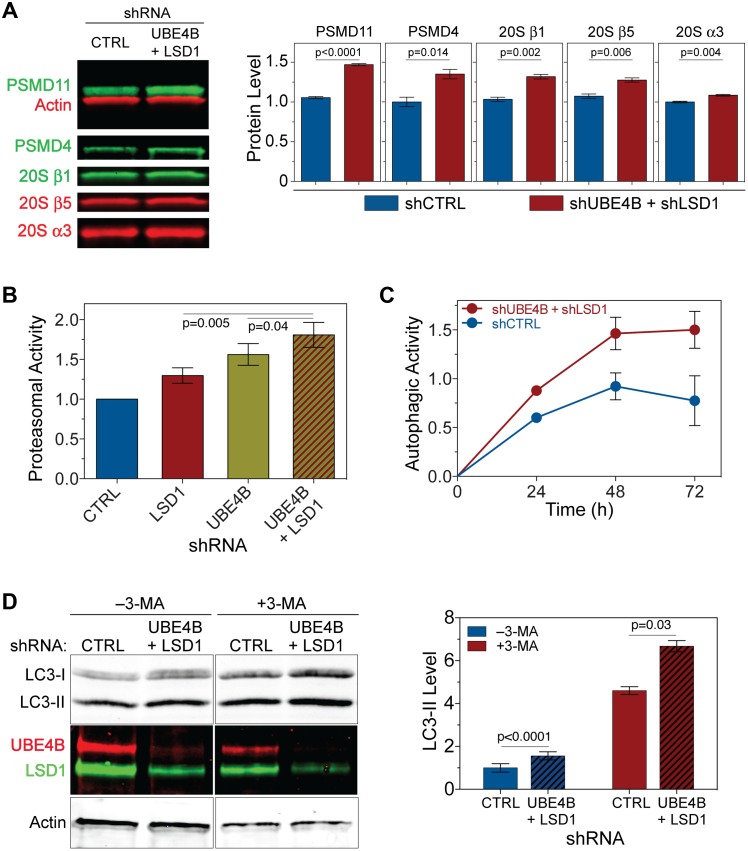
UBE4B and LSD1 double knockdown activates both proteasomes and autophagy. (**A**) Increased protein levels of proteasome subunits upon the UBE4B and LSD1 double knockdown in HEK293T cells. (**B**) The UBE4B and LSD1 single or double knockdowns increase the proteasomal degradation of a reporter substrate, Suc-LLVY-Luciferin, whose degradation is measured in a luciferase release assay. The double knockdown shows synergistic proteasomal activation when compared with the individual knockdowns (*n* = 6). (**C**) The autophagy activity is significantly increased in cells with the UBE4B and LSD1 double knockdown. The in vivo activity of the ATG4B protease, which cleaves LC3 precursors, was quantified via a *Gaussia* luciferase release assay (see S5D Fig., S5E Fig., and Materials and Methods for details). The HCT116 cells were analyzed 48 h after the initiation of the knockdown. (**D**) Quantification of LC3-II levels in HEK293T cells with the UBE4B and LSD1 double knockdown or the mock control. The cells were treated with or without 10 mM 3-methyladenine (3-MA) for 48h before lysis. Western blots indicate an increase in LC3 levels (top panels) in the double UBE4B and LSD1 knockdown cells (middle panels), while the actin control is unchanged (bottom panels). The graph shows the quantification of LC3-II levels as normalized to actin levels (*n* = 3). A one-way ANOVA test with matched multiple comparisons and Tukey correction was used for statistics. Data represent means ± SEM. The numerical data used to make this figure can be found in S1 Data.

To determine whether the enhanced clearance of SOD1^G85R^ upon the knockdown of UBE4B and LSD1 reflects an increase in proteasome-mediated degradation, we examined abundance of several proteasomal subunits. Consistent with our transcriptome analysis that demonstrated an increase in the level of PSMD11 RNA, the PSMD11 protein level was significantly increased in the double-knockdown cells (Fig. 5A). Both PSMD11 and PSMD4 (proteasome 26S subunit, non-ATPase, 4) are resident to the 19S regulatory proteasome particle, and we found that the PSMD4 protein level was also significantly increased. Moreover, components of the 20S core proteasome particle, including 20Sα3, 20Sβ1, and 20Sβ5, were also significantly increased.

To determine whether the increase in the quantities of proteasome subunits corresponds to augmented proteasomal activity, we measured the chymotrypsin-like proteasome activity in lysates derived from HEK293T cells with single or double knockdown of UBE4B and LSD1 using a luciferase assay (S5A Fig.). The proteasomal activity was increased in LSD1 or UBE4B single-knockdown cells, but the highest activity was observed in double-knockdown cells (Fig. 5B). This finding is consistent with the results of *C*. *elegans* suppressor studies and protein solubility assays in mammalian cells, in which both UBE4B and LSD1 are required for the maximal effects of the suppressors. Consistent with the activation of proteasomal activity by the double knockdown of UBE4B and LSD1, inhibition of the proteasome by treating cells with MG132 blocked the degradation of SOD1^G85R^ conferred by the UBE4B and LSD1 knockdown (S5B Fig. and S5C Fig.). Thus, the knockdown of UBE4B and LSD1 significantly increases both the subunit quantity and the activity of proteasomes.

In addition to the activation of the proteasome, we asked whether autophagy is also up-regulated in the UBE4B and LSD1 double-knockdown cells. To measure autophagic activity, we employed a *Gaussia* luciferase (GLuc) release assay [37,38] that reports the autophagy-dependent ATG4B cleavage of an actin-tethered actin-LC3-GLuc-fusion protein and its subsequent release from the cell into the medium (S5D Fig. and S5E Fig.). HCT116 cells, which are amenable to this assay, were transfected with shRNA constructs to knock down UBE4B and LSD1, with the LC3-GLuc plasmid used to measure the cleavage of LC3 and the constitutively secreted control (cytomegalovirus-secreted embryonic alkaline phosphatase [CMV-SEAP]) for transfection/secretion normalization (S5D Fig. and S5E Fig.). The LC3-dependent GLuc activity, measured over a period of 72 h, showed a 2-fold increase in ATG4B proteolytic activity at the end of the time course demonstrating the activation of autophagy by the knockdown of UBE4B and LSD1 (Fig. 5C). The cells transfected with the noncleavable, LC3-less fusion, the Act-GLuc construct, showed only background levels of Gluc activity, similar to the levels observed in nontransfected cells.

To confirm that autophagy was activated by the UBE4B and LSD1 double knockdown, we measured LC3-II accumulation by western blotting in cells. Double knockdown samples showed increased levels of LC3-II compared with controls, even when LC3-II was elevated by treatment with 3-MA (Fig. 5D). Together, these data demonstrate that a UBE4B- and LSD1-dependent protein quality control pathway similar to that in *C*. *elegans* also operates in mammalian cells, since a reduction in these two enzymes promotes the removal of aggregating proteins through enhanced post-translational quality control systems involving the proteasome and autophagy.

### p53 Regulates Protein Quality Control

Until now, p53 has not been associated with antiproteotoxicity activity. p53 has been shown to regulate autophagy, but in opposing directions [39]. Our microarray analysis and subsequent studies establish a correlation between the activation of p53-mediated transcription and enhanced protein quality control conferred by the knockdown of UBE4B and LSD1 (Fig. 5). It has been demonstrated that p53 is a target of polyubiquitination by UBE4B and demethylation by LSD1, and each of these functions decreases the p53 activity [25,28]. Thus, p53 has emerged as a potential effector that mediates the synergistic action of UBE4B and LSD1 in the antiproteotoxicity pathway.

**Fig 6:**
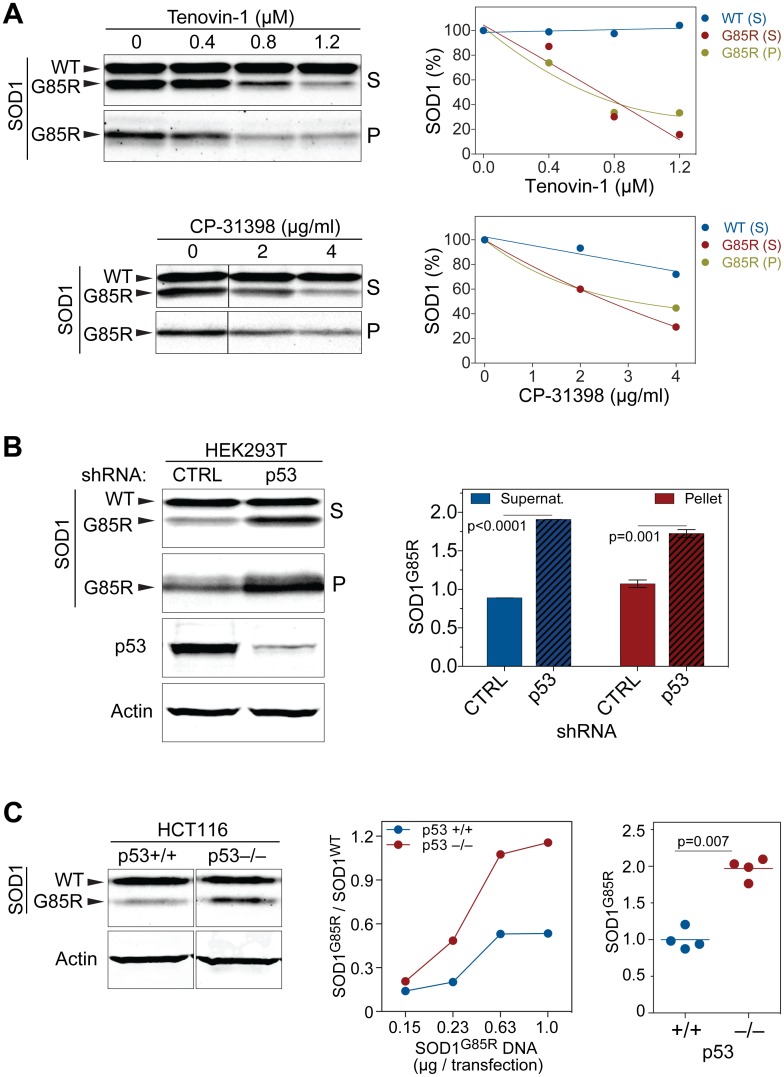
p53 promotes the clearance of misfolded SOD1 mutant proteins. (**A**) p53 small molecule activators Tenovin-1 and CP-31398 reduce the levels of misfolded SOD1 proteins, as determined by the SOD1^G85R^ solubility assay in HEK293 cells. Increasing concentrations of the p53 activators significantly decrease the levels of SOD1^G85R^ but not the endogenous WT SOD1 proteins in western blots of both supernatant and pellet fractions. (**B**) A decrease in p53 as the result of shRNA knockdown increases the levels of SOD1^G85R^ but not WT SOD1 proteins in the SOD1^G85R^ aggregation assay, as shown by western blots of both supernatant (S) (*n* = 2) and pellet (P) (*n* = 3) fractions. (**C**) A complete absence of p53 increases the accumulation of SOD1^G85R^ mutant proteins in p53–/– HCT116 cells when compared with controls. Representative western blots (left panels) and quantification of SOD1^G85R^ levels in the supernatant lysates are shown. The middle graph indicates the ratio of G85R to WT SOD1 proteins in the presence or absence of p53 with varying amounts of transfected mutant SOD1. The right graph panel shows the same data as shown in the middle panel, but normalized to the average SOD1^G85R^ level for each amount of the transfected plasmid. Data represent means ± SEM. The numerical data used to make this figure can be found in S1 Data.

To determine whether p53 directly protects against proteotoxicity, we first used small molecule activators of p53 in the cell-based SOD1^G85R^ protein aggregation assay. Tenovin-1 activates p53 by inhibiting the SIRT1/2 deacetylase and therefore promoting p53 acetylation, thereby increasing its stability and activity [40]. CP-31398 is another drug that activates p53 by stabilizing the p53 DNA-binding domain in an active conformation and inhibiting its ubiquitination [41,42]. Both p53 activators significantly reduced the amount of SOD1^G85R^ protein in the supernatant and pellet fractions (Fig. 6A, S6A Fig., and S6D Fig.) at various concentrations of Tenovin-1 (0.4–1.2 μM) and CP-31398 (2–4 μg/ml). Furthermore, to confirm that Tenovin-1 and CP-31398 acted through the activation of p53, we reduced p53 levels via shRNAs while testing the clearance of SOD1^G85R^ protein with the drug treatment. The p53 reduction blocked the effect of either Tenovin-1 or CP-31398 on promoting SOD1^G85R^ clearance, indicating that the drugs act through p53 (S6G Fig.).

To investigate the mechanism by which the activation of p53 by Tenovin-1 and CP-31398 reduced the levels of SOD1^G85R^ proteins, we asked whether autophagy was activated by these drug treatments. In agreement with a previous report that CP-31398 activates autophagy [43], we observed that increasing concentrations of either Tenovin-1 or CP-31398 up-regulated LC-II protein levels (S6H Fig.), consistent with the activation of autophagy. To further validate that Tenovin-1 and CP-31398 act post-translationally to promote the clearance of SOD1^G85R^ proteins, we performed cycloheximide chase experiments and confirmed the increase in the degradation of SOD1^G85R^ proteins upon treatment of either of the p53 activator drugs (S6B Fig., S6C Fig., S6E Fig., and S6F Fig.).

Conversely, we asked whether reducing p53 activity would negatively affect the clearance of SOD1^G85R^ proteins. First, we performed the SOD1^G85R^ protein solubility assay in cells in which p53 was reduced by RNAi. We found that partial removal of p53 in HEK293T increased SOD1^G85R^ protein levels in both the supernatant and pellet fractions (Fig. 6B). Next, using a human HCT116 cell line in which p53 was knocked out, we asked how the complete removal of p53 affected the clearance of misfolded SOD1^G85R^. Unlike the WT SOD1 protein, whose level was not affected by the absence of p53, the SOD1^G85R^ mutant protein was significantly increased in the p53 knockout cells when compared with the controls, indicating that endogenous p53 promotes the clearance of misfolded proteins (Fig. 6C).

**Fig 7:**
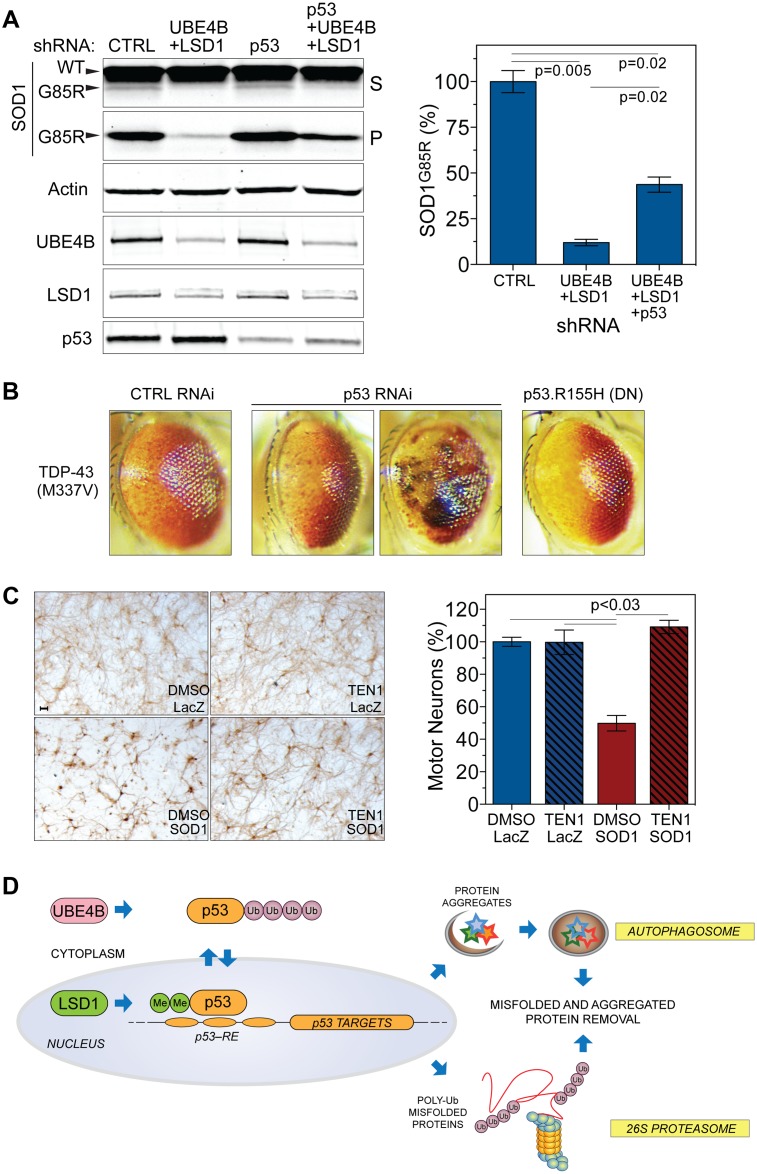
Enhanced protein quality control by the knockdown of UBE4B and LSD1 depends on p53. (**A**) Left: p53 knockdown reverses the enhanced clearance of SOD1^G85R^ proteins conferred by the UBE4B and LSD1 double knockdown. Total amounts of shRNAs were adjusted to be equal with nontargeting CTRL shRNAs. Right: quantification of insoluble aggregated SOD1^G85R^ in pellet fractions from HEK293T cells transfected with control, double (UBE4B/LSD1), or triple (UBE4B/LSD1/p53) shRNAs (*n* = 2). (**B**) The degenerative TDP-43^M337V^ eye phenotype is exacerbated by the knockdown of the *Drosophila* homolog of p53 (p53 RNAi), or by the overexpression of a dominant-negative p53 mutant (p53.R155H). Expression of p53 RNAi, p53.R155H, and TDP-43^M337V^ are driven by GMR-Gal4. (**C**) The p53-activating drug Tenovin-1 (TEN1) protects spinal cord motor neurons from SOD1^G85R^-induced proteotoxicity. Rat spinal cord cultures were grown as described in Materials and Methods and treated with 0.8 μM TEN1 or vehicle (VEH) DMSO, followed by infection of HSV-SOD1^G85R^ or the HSV-LacZ control after 24 h. At day 5 post-infection, cells were fixed and stained with a motor neuron-specific anti-neurofilament H (NF-H) antibody. Left: representative images of motor neurons in each condition. Scale bar = 50 μm. Right: quantification of motor neuron survival (one-way ANOVA with multiple comparisons and Tukey correction). Data represent means ± SEM. (**D**) Schematic of the SUNS pathway. A reduction in UBE4B and LSD1 function synergistically activates the p53-mediated transcriptional program, promoting clearance of misfolded and aggregated proteins via increased proteasomal and autophagic clearance. The numerical data used to make this figure can be found in S1 Data.

To determine whether p53 mediates the UBE4B- and LSD1-dependent clearance of the SOD1^G85R^ proteins, we knocked down UBE4B and LSD1 with or without the removal of p53 and then examined the levels of SOD1^G85R^. We applied both transient and stable shRNA knockdown by creating an inducible, stable HEK293T cell line expressing tetracycline-regulated shRNAs against UBE4B, LSD1, and p53. Both transient and stable knockdown of p53 significantly reversed the SOD1^G85R^ protein clearance conferred by the UBE4B and LSD1 knockdown (Fig. 7A and S7A Fig.). This result was confirmed with an independent set of shRNAs against UBE4B and LSD1 (S7B Fig.) and by knockdown of UBE4B and LSD1 in HCT116 cells with or without the p53 gene knocked out (S7C Fig.). Notably, unlike HEK293T cells, HCT116 cells do not express SV40 large T-antigen (LT-Ag), a regulator of p53 stability [44], suggesting that the action of UBE4B and LSD1 does not require LT-Ag. Taken together, these results demonstrate that p53 is required for the UBE4B- and LSD1-dependent clearance of the SOD1^G85R^ proteins, and it acts downstream of UBE4B and LSD1 to positively regulate the clearance of misfolded proteins.

To confirm that p53 can modulate proteotoxicity in vivo, we used the *Drosophila* TDP-43^M337V^ neurodegeneration model as described earlier (Fig. 2D) [22]. Either knockdown of p53 by RNAi or expression of a dominant negative form of *Drosophila* p53 (p53.R155H) [45] with the GMR-Gal4 driver exacerbated the TDP-43^M337V^-induced eye phenotype (Fig. 7B). This aggravation of the phenotype was evident in increased loss of pigmented ommatidia and, in p53 RNAi flies, the appearance of necrotic patches, which were observed at low penetrance (Fig. 7B). Expression of the dominant negative p53.R155H transgene on its own, in a wild-type background, did not cause any eye phenotype. Together, these results indicate that endogenous p53 plays a role in reducing TDP-43^M337V^ proteotoxicity in the *Drosophila* eye.

We further tested whether p53 activation would alleviate SOD1^G85R^-induced neurotoxicity. We employed a previously characterized SOD1 neurotoxicity assay [46], in which spinal cord primary motor neurons were prepared from rat embryos, maintained on astrocyte monolayers supplemented with neurotrophic factors, and stained with a mature motor neuron marker, the neurofilament H (NF-H) antibody SMI-32. Expression of SOD1^G85R^ via a neuron-specific herpes simplex virus (HSV) vector reduced the survival of motor neurons significantly by approximately 50% in contrast to the HSV-LacZ control over 5 days (Fig. 7C). When treated with the p53 activator Tenovin-1, the motor neurons showed protection from SOD1^G85R^-induced proteotoxicity, as compared with the vehicle control. Among the various concentrations tested, 0.8 μM of Tenovin-1 completely blocked the neurotoxicity of SOD1^G85R^, with minimal toxicity from the drug itself (Fig. 7C). These results confirm that the activation of p53 provides protection against the toxicity of misfolded proteins in neurons.

## Discussion

This study uncovers a previously unknown pathway that mitigates the toxicity of misfolded proteins by boosting protein quality control systems. Using a *C*. *elegans* genetic screen for suppressors of neurotoxicity induced by mutant SOD1, we have identified the SUNS pathway, which is mediated by two conserved genes, *ufd-2*/UBE4B and *spr-5*/LSD1. From *C*. *elegans* to human cells, inactivation of the highly conserved lysine-modifying enzymes *ufd-2*/UBE4B and *spr-5*/LSD1 is shown to enhance the clearance of misfolded proteins. In mammalian cells, the pathway mediated by UBE4B and LSD1 acts to improve the cellular protein quality control by increasing proteasomal and autophagic activities (Fig. 7D). Although it was initially surprising that loss of ubiquitin ligase UBE4B and lysine-specific demethylase LSD1 protects against proteotoxicity, further results reveal positive downstream effectors, including transcription factors, with a novel implication of p53 in antiproteotoxicity activities. Together, these results demonstrate the capacity of a cell to reprogram its protein quality control through transcriptional regulation to defend against proteotoxicity.

### A Protein Quality Control Pathway Regulated by the Lysine-Modifying Enzymes UBE4B and LSD1

We isolated the SUNS *C*. *elegans* mutant based on the potent suppression of SOD1-induced neurotoxicity. The suppressor was found to significantly enhance the removal of misfolded proteins, underscoring the critical role of protein misfolding in SOD1-mediated neurodegeneration. The enhanced clearance also applies to other misfolded proteins, such as TDP-43, FUS, and polyglutamine-containing proteins, indicating a general improvement in protein quality control. This rare but strong suppressor requires modulation of only two genes, suggesting that it provides a major protein quality control program with a readily accessible switch. Furthermore, the synergistic cooperation of two genes, *ufd-2* and *spr-5*, points to a common downstream pathway with integrative regulation.

Consistent with the observation that the loss of function of *C*. *elegans ufd-2* and *spr-5* promotes the clearance of misfolded proteins, inactivation of their *Drosophila* and mammalian orthologs reduces the toxicity of aggregation-prone proteins, indicating the existence of a protein quality control regulatory mechanism that is functionally conserved across species. Interestingly, both mammalian genes encode lysine-modifying enzymes: UBE4B is a U-box type ubiquitin ligase, and LSD1 is a lysine-specific protein demethylase. Both UBE4B and LSD1 are highly expressed in neurons and essential for early development in mammals [47–50].

In contrast to the conventional notion that ubiquitin ligase promotes protein degradation, our studies indicate that UBE4B negatively affects the clearance of misfolded proteins, and its down-regulation protects against severe proteotoxicity. In line with our observation that the down-regulation of UBE4B protects against proteotoxicity in the nervous systems of *C*. *elegans* and *Drosophila*, mice with elevated levels of UBE4B show autophagy defects with accumulation of ubiquitin- and p62-positive aggregates in the brain [51]. UBE4B forms a complex with an AAA-ATPase p97/VCP to ubiquitinate and degrade specific client proteins [47,52]. p97/VCP plays an essential role in handling unfolded proteins, such as in endoplasmic-reticulum-associated protein degradation [53], and it was recently linked to familial ALS [54]. Our findings thus provide a new link between p97/VCP and protein quality control, which is regulated by UBE4B.

The fact that both UBE4B and LSD1 are enzymes catalyzing post-translational modifications suggests that their effects on protein quality control can be timely, energy-efficient, and integrative. The synergistic interaction between the two lysine-modifying enzymes, UBE4B and LSD1, also suggests that their downstream pathways converge to influence protein quality control. Consistent with recent studies showing enhancement of protein quality control [20,55–57], the identification of the strong antiproteotoxic effects mediated by UBE4B and LSD1 demonstrates that plasticity of the cellular protein quality control programs can be substantially augmented to yield overall protection to an organism.

### p53 as a Key Switch in Protein Quality Control

Unbiased transcriptome analysis points to p53 as a central regulator of the transcriptional reprograming that mediates the effects of UBE4B and LSD1 on protein quality control. Consistent with this observation, p53 has been found to have a number of direct transcriptional targets functioning in protein quality control and neuroprotection, and it also activates additional stress-response transcription factors such as FOXOs [33]. Interestingly, p53 is elevated in the central nervous system of patients with neurodegenerative conditions such as Alzheimer disease and ALS [58,59]. Our observation that the transcription factors mediate the effects of this strong suppressor is reminiscent of other signaling pathways governing protein homeostasis. For example, the heat shock response activates the expression of molecular chaperones and other protein quality control machinery via the master transcription regulators, the heat shock factors [60]. Also, the unfolded protein response promotes the endoplasmic reticulum (ER) quality control programs through the activation of a set of the transcription factors, including XBP1, ATF4, and ATF6 [61]. In recurring themes, the post-translational regulation by UBE4B and LSD1 activates the p53 transcription factor, which is then capable of eliciting a systematic protective program against proteotoxic stress.

p53 has a well-established role in regulating responses to DNA damage [62,63], and recently, a neuroprotective role of an activated DNA damage checkpoint has been demonstrated in a tau-dependent neurodegeneration model [64]. Here we propose that p53 is a versatile transcriptional switch that guards against both genotoxicity and proteotoxicity. The specific activity of p53 may be fine-tuned at the post-translational level by upstream regulators such as UBE4B and LSD1. In addition, it is known that p53 promotes apoptosis in cells with irreversible genotoxic damage [65]. p53 may also function as a dual regulator in proteotoxicity: it promotes the repair and survival of moderately damaged cells but turns on cell death pathways in cells whose damage is irreparable. Such duality has been observed for other protein quality control systems, such as the ER stress responses [61]. Thus, p53 could serve as a critical regulator of cellular responses to proteotoxicity by repairing or removing damaged cells.

Taken together, these findings reveal a previously unrecognized pathway that systematically antagonizes the proteotoxicity associated with neurodegenerative diseases, and they point to potential targets for harnessing the protective capacity of the cells’ reprogrammed protein quality control to develop a wide-spectrum antiproteotoxicity therapeutic strategy.

## Materials and Methods

### Ethics Statement

The pregnant rat dams were euthanized by overdosing with nembutal. The Children’s Hospital of Philadelphia IACUC approved these procedures (protocol #597).

### DNA Plasmids

For mammalian expression, SOD1 and TDP-43 were expressed in pEF-BOS and pRK5-Myc, respectively, as previously described [9,20]. The UBE4B (TF308519) and LSD1 shRNA (TF316984) plasmids and the scrambled control (TR30015) were from Origene. The p53 shRNA plasmid pLVTH-sip53 and control pLVTH were from D. Trono (Addgene #12239) [66]. The p53 transcriptional reporter PG13-Luc was a generous gift from B. Vogelstein [67]. The autophagy luciferase release plasmids Act-LC3-Gluc and Act-Gluc were kindly provided by B. Seed [37], and the control pCMV-SEAP was from A. Cochrane (Addgene #24595). For transgenic *C*. *elegans*, *ufd-2* and *spr-5* complementary DNAs (cDNAs) were cloned into a vector under the control of an *snb-1* promoter, as previously described [20]. Additional information on the shRNA targeting sequences and vectors is given in the Supporting Information Materials and Methods (S1 Text).

### *C*. *elegans* Strains, Suppressor Screen, and Mutation Identification

The Bristol N2 *C*. *elegans* strain was used in all experiments unless otherwise specified. A list of *C*. *elegans* strains is given in the Supporting Information Materials and Methods (S1 Text). Transgenic lines were generated according to standard procedures by injecting 20 μg/ml of expression plasmid DNA into hermaphrodite gonads. For the suppressor screen, worms were mutagenized with 47 mM ethyl methanesulfonate, and a semiclonal strategy was used with five P0 worms in one plate. Suppressors were visually selected based on strong recovery in the movement phenotype in the F2 generation. The suppressor mutations were mapped by using single-nucleotide polymorphism markers in the Hawaiian strain and then identified by whole-genome deep sequencing, followed by Sanger sequencing validations (see Supporting Information Materials and Methods [S1 Text]).

### *C*. *elegans* Locomotor Assay and Microscopy

The *C*. *elegans* strains were observed stereoscopically, and their motility was quantified by the thrashing assay [20]. Animals were transferred from the feeding plate into M9 buffer (3 mg/ml KH_2_PO_4_, 6 mg/ml Na_2_HPO_4_, 5 mg/ml NaCl and 1 mM MgSO_4_). After 1 min of adaptation, the number of body bends or thrashes was counted for 1 min as an index of the locomotor phenotype. A thrash was counted when both the head and the tail bent away from the anteroposterior axis by more than 45°. Videos of *C*. *elegans* locomotion were recorded using a Leica M165 fluorescence stereoscope. High-magnification imaging was performed on a Zeiss AxioObserver Z1 with Apotome, with *C*. *elegans* immobilized by 10 mM levamisole.

### *Drosophila* Strains and Assays

See Supporting Information Materials and Methods (S1 Text).

### Mammalian Cell Lines, Transfections, Antibodies, and Drug Treatments

See Supporting Information Materials and Methods (S1 Text).

### Protein Solubility Assay

The protein solubility assay to measure aggregate proteins in *C*. *elegans* and mammalian cells was modified from a previously described protocol [9] (see Supporting Information Materials and Methods [S1 Text]).

### Transcriptional Activity Luciferase Assay

After a 72-h knockdown, cells were detached and transfected with firefly luciferase p53 reporter plasmid (PG13-luc), together with a thymidine kinase promoter *Renilla* luciferase (tk-Rluc) reporter for normalization. Cells were lysed in passive lysis buffer (Promega) 24 h after transfection and analyzed with the Dual Luciferase Reporter System according to the manufacturer’s recommendations (Promega) using an injector-equipped Synergy H1 microplate reader (Bio-Tek).

### Proteasome Activity Assay

Proteasome assays were performed as described previously [68], using the Suc-LLVY-Luciferin substrate for chymotrypsin-like activity of the proteasome (the Proteasome Glo kit, Promega). In brief, cells were detached and washed in DMEM/10, followed by several washes in cold PBS. Proteasome lysis buffer (50 mM Tris-HCl, pH 7.5, 0.025% digitonin, 250 mM sucrose, 5 mM MgCl_2_, 0.5 mM EDTA, 2 mM ATP, and 1 mM DTT) was added to the cells and incubated on ice for 5–10 min. The lysates were then centrifuged for 15 min at 20,000 *g* to isolate the cytoplasm containing the proteasomes. The supernatant was transferred to a fresh tube, and equal amounts of protein were used in each assay.

### Autophagy Assays

Autophagy was quantified with a *Gaussia* luciferase release assay [37,38], which is based on the ATG4B-induced proteolytic cleavage of an actin-anchored fusion LC3-Gluc fusion protein (S5D Fig. and S5E Fig.). ATG4B-induced proteolytic cleavage of LC3 releases the Gluc fragment and enables its secretion into the cell medium. The activity of the released Gluc in the medium (together with constitutively secreted SEAP) was measured by the Secrete-Pair Dual Luminescence Assay kit (GeneCopoeia). Cells, plated in 12-well dishes, were transfected with the Act-LC3-Gluc or control Act-Gluc plasmid together with the normalization control, CMV-SEAP. At 24 h after transfection, the DMEM/10 medium was replaced, and 100 μl of cell growth medium was withdrawn at 24 h, 48 h, and 72 h. The medium was centrifuged at 6,000 *g* for 5 min to remove detached cells, followed by the luciferase analysis according to the manufacturer’s recommendations (GeneCopoeia) using a microplate reader (Synergy H1, Bio-Tek).

For LC3 western blot analysis, cells were lysed in LC3 buffer (50 mM Tris-Cl, pH 8.0, with 1% SDS, 0.5% NP40, 150 mM NaCl, and 5 mM EDTA) and sonicated with a Diagenode Bioruptor device (set on high, 30-sec pulse, 30-sec pause, 7.5 min total).

### Microarray Transcriptional Profiling and Quantitative RT-qPCR

Total RNA was isolated from HEK293T cells with the RNeasy Mini kit and analyzed using the Affymetrix human GENE 1.0ST array. The microarray data were managed using the Partek Genomic Suite (Partek, St. Louis) and Spotfire DecisionSite software (TIBCO Software, Palo Alto, California) and analyzed using Ingenuity Pathways Analysis software (IPA, Ingenuity Systems). In addition to RNA, total protein was isolated from the same samples by acetone precipitation and resolubilizing the flow-through lysates to verify the reduction of the UBE4B and LSD1 proteins. For quantitative RT-qPCR validations, cDNAs were synthesized with the QuantiTect reverse transcription kit (Qiagen). Primers for quantitative RT-qPCR were from PrimerBank (S3 Table) [69]. RT-qPCRs were performed on a BioRad thermal cycler with iQ SYBER Green PCR mix (BioRad).

### Spinal Motor Neuron Survival Assay

Embryonic Sprague Dawley rat spinal cord cultures and neuronal survival assays were previously described [46] (see Supporting Information Materials and Methods [S1 Text]).

### Statistical Analysis

The *p*-values for all analyses were obtained using Student’s *t* tests performed in Excel or GraphPad Prism 6, unless otherwise indicated. For the microarray data, Student’s *t* test was used to analyze the gene expressions. For the Upstream Regulator Ingenuity Pathway Analysis, Fisher’s exact test was used. For the LC3-II western blotting analysis and the spinal cord motor neuron toxicity assay, a one-way ANOVA with multiple comparison test was used.

## Supporting Information

S1 Data Numerical data used in figure preparation. (XLSX)

S1 Fig The levels of misfolded proteins in *C*. *elegans* and *Drosophila* models. (**A**) A schematic for protein misfolding and aggregation in relation to protein solubility and quality control. For an aggregation-prone protein, there is a dynamic equilibrium among correctly folded native proteins, its misfolded forms, oligomeric aggregates, and large aggregates. The relative proportion of these species depends on the intrinsic folding properties of the protein and the cellular environment of protein quality control. In a protein solubility assay, large aggregates can be sedimented into the insoluble fraction via extraction and centrifugation, while smaller oligomeric aggregates and other misfolded proteins are retained in the soluble fraction. The misfolded and aggregated proteins are targets of cellular protein quality control machineries including molecular chaperones, the ubiquitin-proteasome system, and autophagy. (**B**) Western blotting analyses of *C*. *elegans* indicate that the *ufd-2* and *spr-5* mutations do not substantially alter the total protein levels of untagged SOD1^G85R^. The western blot lanes are from the same gel and exposure. (**C**) Total protein levels of YFP-tagged SOD1^G85R^, TDP-43^c25^, or PolyQ proteins are reduced in double *ufd-2* and *spr-5* mutant strains, suggesting that larger fractions of YFP-tagged misfolded proteins are removed in the mutant strains. (**D**) Total protein levels of untagged TDP-43^M337V^ or FUS^R521C^ are not substantially altered in *Drosophila* with the knockdown of *ufd-2*/UBE4B (CG9934) or *spr-5*/LSD1 (Su(Var)3-3).
(TIF)

S2 Fig Knockdown of UBE4B and LSD1 promotes the degradation of SOD1^G85R^ and TDP-43^Q331K^ proteins in mammalian cells. (**A**) The flow chart of the mammalian cell-based protein solubility assay as described in the Materials and Methods. (**B**) A representative western blot of total SOD1^G85R^ protein in HEK293T cells treated with UBE4B and LSD1 shRNAs. (**C**) Quantification of total SOD1^G85R^ protein and its corresponding supernatant (S) and pellet (P) fractions. The quantification is based on the band intensities and the relative amounts of S and P fractions as parts of the total lysate that were loaded on the gel. The percentage of insoluble SOD1^G85R^ protein in the total SOD1^G85R^ protein remains relatively stable at 25%–30%. (**D**) A representative western blot of the TDP-43^Q331K^ protein solubility assay. HEK293T cells were transfected with a TDP-43^Q331K^ expression plasmid, together with a control shRNA (CTRL) or mixed UBE4B and LSD1 shRNA plasmids. Following cell lysis and fractionation, S and P fractions were run on 15% SDS-PAGE gels. The western blot lanes are from the same gel and exposure. (**E**) Quantification of TDP-43^Q331K^ in the S and P fractions, containing smaller and larger aggregates, respectively, shows a significant reduction in protein levels caused by the UBE4B and LSD1 knockdown (*n* = 2 for S, *n* = 3 for P). (**F**) Western blot analyses of cycloheximide chase show that TDP-43^Q331K^ protein is degraded faster in double UBE4B and LSD1 knockdown cells than in the nontargeting shRNA control cells. (**G**) Quantification of chase experiments in (F) (*n* = 2). Data represent means ± SEM. The numerical data used to make this figure can be found in S1 Data.
(TIF)

S3 Fig The transcriptional profiling and network analysis. (**A**) The heat map of microarray signals of differentially regulated genes (*p* < 0.05) upon the knockdown of LSD1 alone, UBE4B alone, or both, in triplicates. Hierarchical clustering of the samples indicates that the single UBE4B knockdown induces similar transcriptional changes as the double knockdown, consistent with the pattern of antiproteotoxic activities shown in Fig. 3A and 3B. (**B**) The heat map of p53 transcriptional targets. The hierarchical clustering of the samples demonstrates the same pattern as shown above for all differentially regulated genes. (**C**) The p53 network is activated in the UBE4B and LSD1 double-knockdown cells (p53: z-score = 2.0; *p*-value of overlap = 2.49 x 10^-2^). The transcriptional targets with changes consistent with p53 activation are shown, with up-regulated genes in red and down-regulated genes in green.
(TIF)

S4 Fig UBE4B and LSD1 regulate p53 and its downstream factor FOXO. (**A**) Elevation of MDM2, the p53-targeting ubiquitin ligase, significantly reduced the p53 response element-mediated activity that was induced by the UBE4B and LSD1 knockdown (*n* = 5). MDM2 is co-transfected with the p53 reporter upon knockdown of UBE4B and/or LSD1. (**B**) The dimethylation at the K370 residue of the p53 protein (Me_2_-K370-p53) is increased in double UBE4B and LSD1 knockdown cells. In HEK293T cells treated with UBE4B and LSD1 shRNAs, there is an increase of Me_2_-K370-p53 relative to the total p53 protein in the nucleus-enriched fraction as shown in the western blots (left) and quantification chart (right, *n* = 3). (**C**) A constitutively active forkhead responsive element luciferase reporter (FOXO1-AAA) was used to demonstrate that the knockdown of UBE4B and LSD1 specifically induces the FOXO1 transcriptional activity (*n* = 3). (**D**) *C*. *elegans* locomotion assays indicate the rescuing effects of loss-of-function mutations *spr-5(by134)*;*ufd-2(tm1380)* (indicated by-/-) on the neurotoxicity of transgenic SOD1^G85R^ protein. Loss-of-function mutation *daf-16(mu86)* partially reversed locomotion rescue of *spr-5(by134)*;*ufd-2(tm1380)* mutations (*n* > 40). Data represent means ± SEM. The numerical data used to make this figure can be found in S1 Data.
(TIF)

S5 Fig The proteasomal and autophagic activity assays. (**A**) A schematic of a luciferase-based proteasomal activity assay. Isolated cytosol is mixed with a peptide substrate, Suc-LLVY-luciferin. The chymotrypsin-like activity of proteasomes cleaves off the Suc-LLVY peptide, releasing the amino-luciferin, which produces strong chemiluminescence in the presence of luciferase and ATP. The detected chemiluminescence is used to quantify the proteasomal activity. (**B**) Inhibition of the proteasomal activity reverses the reduction of insoluble SOD1^G85R^ protein in the pellet fraction induced by the UBE4B and LSD1 knockdown. Western blots are shown for insoluble SOD1^G85R^ proteins from MG132-treated (20 μM) or untreated (DMSO) HEK293T cells with knockdown of UBE4B and LSD1 or nontargeting shRNA controls. Cells were treated with MG132 for 48 h and lysed 72 h post-transfection. (**C**) Quantification of SOD1^G85R^ levels from western blots as shown in (B), *n* = 2. Data represent means ± SEM. (**D**) The flow chart of an autophagic activity assay to measure LC3 cleavage based on a luciferase (GLuc) reporter. Cells were transfected with a set of plasmids to knockdown LSD1 and UBE4B (or nontargeting shRNA, CTRL) and to express the GLuc reporters and SEAP (secreted embryonic alkaline phosphatase). SEAP is constitutively secreted and serves as a transfection normalization control. SOD1^G85R^ is expressed concurrently to match the condition with the increased burden of misfolded proteins, as described earlier (Fig. 3). (**E**) A schematic of the LC3 cleavage and Gluc release assay. A cleavable fusion protein, Actin(Act)-LC3-GLuc, or its uncleavable negative control, Act-GLuc, is anchored to the actin cytoskeleton inside the cell. When Act-LC3-GLuc is cleaved by the autophagy-associated protease ATG4B, the GLuc fragment is released from its actin anchor and rapidly secreted out of the cell. The activity of GLuc in the cell medium is assayed over a period of several days using the Dual Luminescence Assay kit. The numerical data used to make this figure can be found in S1 Data.
(TIF)

S6 Fig p53-activating drugs enhance the clearance of misfolded mutant SOD1 proteins. (**A**) The p53 small molecule activator, Tenovin-1 (TEN1, 2 μM), significantly decreased the levels of misfolded SOD1^G85R^ but not WT SOD1 proteins in both supernatant and pellet fractions as compared with vehicle-treated controls (VEH) (*n* = 4). Representative western blots are shown in Fig. 6A. The SOD1^G85R^ solubility assay in HEK293T cells is described in Materials and Methods. (**B**) Western blots of cycloheximide chase experiments with Tenovin-1-treated or untreated HEK293T cells. (**C**) Quantification of the chase experiments shows increased SOD1^G85R^ degradation in Tenovin-1-treated cells (*n* = 2). (**D**) Another p53 small molecule activator, CP-31398 (4 μg/ml), also significantly decreased the levels of misfolded SOD1^G85R^ proteins but not WT SOD1 proteins in both supernatant and pellet fractions (*n* = 3). Representative western blots are shown in Fig. 6A. (**E**) Western blots of cycloheximide chase experiments with CP31398-treated or untreated HEK293T cells. (**F**) Quantification of the chase experiments shows increased SOD1^G85R^ degradation in CP31398-treated cells (*n* = 2). Data represent means ± SEM. (**G**) Knockdown of p53 blocked the improved clearance of misfolded SOD1^G85R^ proteins by Tenovin-1 or CP-31398. HEK393T cells were transfected with SOD1^G85R^ and treated with p53-activating drugs Tenovin-1 or CP31398. Protein aggregation assays were performed to evaluate the levels of SOD1^G85R^ proteins in the supernatant (S) and pellet (P) fractions. The reduction of SOD1^G85R^ aggregation in cells treated with Tenovin-1 or CP-31398 is dependent on p53, as the knockdown of p53 abolishes the ability of the drugs to remove aggregates. (**H**) p53-activating drugs activate autophagy as indicated by LC3 protein levels. HEK293T cells treated with Tenovin-1 or CP-31398 for 24 h were lysed in 1% SDS buffer, and LC3-I and LC-II levels were analyzed by western blots. For both Tenovin-1 and CP-31398, the LC-II levels are augmented with increasing drug concentrations. Both panels were from the same gel and western blot with identical imaging settings. The numerical data used to make this figure can be found in S1 Data.
(TIF)

S7 Fig p53 mediates improved protein clearance induced by the knockdown of UBE4B and LSD1. (**A**) Stable knockdown of p53 partially blocks the improved clearance of SOD1^G85R^ proteins conferred by the knockdown of UBE4B and LSD1. A stable cell line with inducible knockdown of p53 via an integrated shRNA (in a pR4R3-TET-PURO vector) is used to conditionally remove p53 upon the induction of doxycycline (DOX). The protein solubility assay was used to analyze the SOD1^G85R^ protein levels in supernatant (S) and pellet (P) fractions. The knockdown of UBE4B and LSD1 substantially enhances the clearance of misfolded SOD1^G85R^ proteins, but this effect is partially reversed by the DOX-induced knockdown of p53 (+DOX). The western blots for the S and P fractions are from the same gels. (**B**) Transient knockdown of p53 blocks the improved clearance of SOD1^G85R^ proteins conferred by the knockdown of UBE4B and LSD1. The protein solubility assay was performed as in (A), except with the knockdown of p53 achieved through shRNA transient transfection and with LSD1 and UBE4B shRNAs in a pR4R3-NEO vector targeting their respective 3′ UTRs (see Materials and Methods). (**C**) Loss of p53 blocks increased SOD1^G85R^ protein clearance conferred by the knockdown of UBE4B and LSD1 in HCT116 cells. HCT116 cells with either the p53 knockout or WT genotype were treated with the UBE4B and LSD1 shRNAs or nontargeting controls. Left: western blot analyses of the insoluble SOD1^G85R^ proteins show that loss of p53 increases the levels of the mutant SOD1 proteins, and GAPDH is a loading control. Right: quantification of the western blots indicates that p53 is required for the effect of UBE4B and LSD1 on SOD1^G85R^ protein clearance (*n* = 3). Data represent means ± SEM. The numerical data used to make this figure can be found in S1 Data.
(TIF)

S1 Movie The SUNS mutant M1 significantly suppresses severe locomotor defects in the *C*. *elegans* model of ALS expressing SOD1^G85R^ in neurons. An SOD1^G85R^-expressing *C*. *elegans* with severe locomotor defects is shown side by side with an isolated suppressor mutant (M1) that exhibits significantly improved movement. The animals were age matched and videotaped as day 1 adults.
(MP4)

S1 Table Upstream regulator analysis of microarray transcriptional profiles upon LSD1 and UBE4B single or double knockdowns. The human transcriptome microarray analysis was carried out in triplicates of four conditions, control shRNA (CTRL), LSD1 knockdown alone, UBE4B knockdown alone, and UBE4B+LSD1 double knockdown, all in the presence of misfolded SOD1^G85R^, as described in the Results and the Materials and Methods. This table contains six sheets (A–F) that summarize the upstream regulator analysis. (**A**) Activated upstream regulators with z-score ≥ 2.0 for each of the three knockdown conditions, LSD1 alone, UBE4B alone, and (UBE4B+LSD1), as compared with the control. A z-score over 2.0 indicates that the particular upstream regulator is significantly activated because its target genes are differentially regulated accordingly in the experimental dataset. Note that the expression levels of the upstream regulators are often not changed. (**B**) Upstream regulators in the double UBE4B+LSD1 knockdown cells, with stringent *p*-values of overlap (*p* < 0.005) but any z-score. Activation/inhibition of upstream regulators with z-score between -2.0 and +2.0 is considered statistically nonsignificant and therefore not predicted. Upstream regulators with z scores ≥2.0 and ≤–2.0 are considered in an “activated” or “inhibited” state, respectively. (**C**) Expression analysis of 130 p53 target genes that are differentially regulated in the UBE4B and LSD1 double knockdown compared with the control. 49 “activated” genes are those whose actual changes are in agreement with the predicted direction in the event of p53 activation. 35 “inhibited” genes are those whose actual changes are opposite to the predicted direction in the event of p53 activation. Another 44 “affected” genes are those that are differentially regulated in the experiment but without IPA-predicted directions. (**D, E, F**) Lists of all upstream regulators in each of the three knockdown conditions, LSD1 alone, UBE4B alone, and both, as compared with the control.
(XLSX)

S2 Table The full microarray datasets comparing control with LSD1 and UBE4B single or double knockdowns. (XLSX)

S3 Table Primer sequences for RT-qPCR reactions. (XLSX)

S1 Text Supporting Information Materials and Methods. (DOC)

## Abbreviations

3-MA: 3-methyladenine
Act: actin
ALS: amyotrophic lateral sclerosis
AOL: amine oxidase-like
cDNA: complementary DNA
CMV: cytomegalovirus
EMS: ethyl methanesulfonate
ER: endoplasmic reticulum
FHRE: forkhead-responsive element
FOXO: forkhead box O
FTD: frontotemporal dementia
FUS: fused in sarcoma
GFP: green fluorescent protein
Gluc: *Gaussia* luciferase
GMR: glass multimer reporter
HSV: herpes simplex virus
IPA: Ingenuity Pathway Analysis
LC3: microtubule-associated protein 1, light chain 3 protein
LSD1: lysine-specific demethylase 1
LT-Ag: large T-antigen
Luc: luciferase
NF-H: neurofilament H
P: pellet fraction
polyQ: polyglutamine
PQC: protein quality control
RNAi: RNA interference
RT-qPCR: reverse transcription-quantitative polymerase chain reaction
S: soluble fraction
SEAP: secreted embryonic alkaline phosphatase
SEM: standard error of the mean
shRNA: small hairpin RNA
SNP: single-nucleotide polymorphism
SOD1: superoxide dismutase
SUNS: *spr-5-* and *ufd-2*-dependent neurodegeneration suppressor
TDP-43: TAR DNA binding protein 43
UBE4B: ubiquitination factor E4 B
WT: wild type
YFP: yellow fluorescent protein
