# Supplementary figures and images for: Regulation of Protein Quality Control by UBE4B and LSD1 through p53-Mediated Transcription
Source: PLoS Biol. 2015 Apr 2;13(4):e1002114. doi: 10.1371/journal.pbio.1002114 (PMC4383508; doi:10.1371/journal.pbio.1002114)

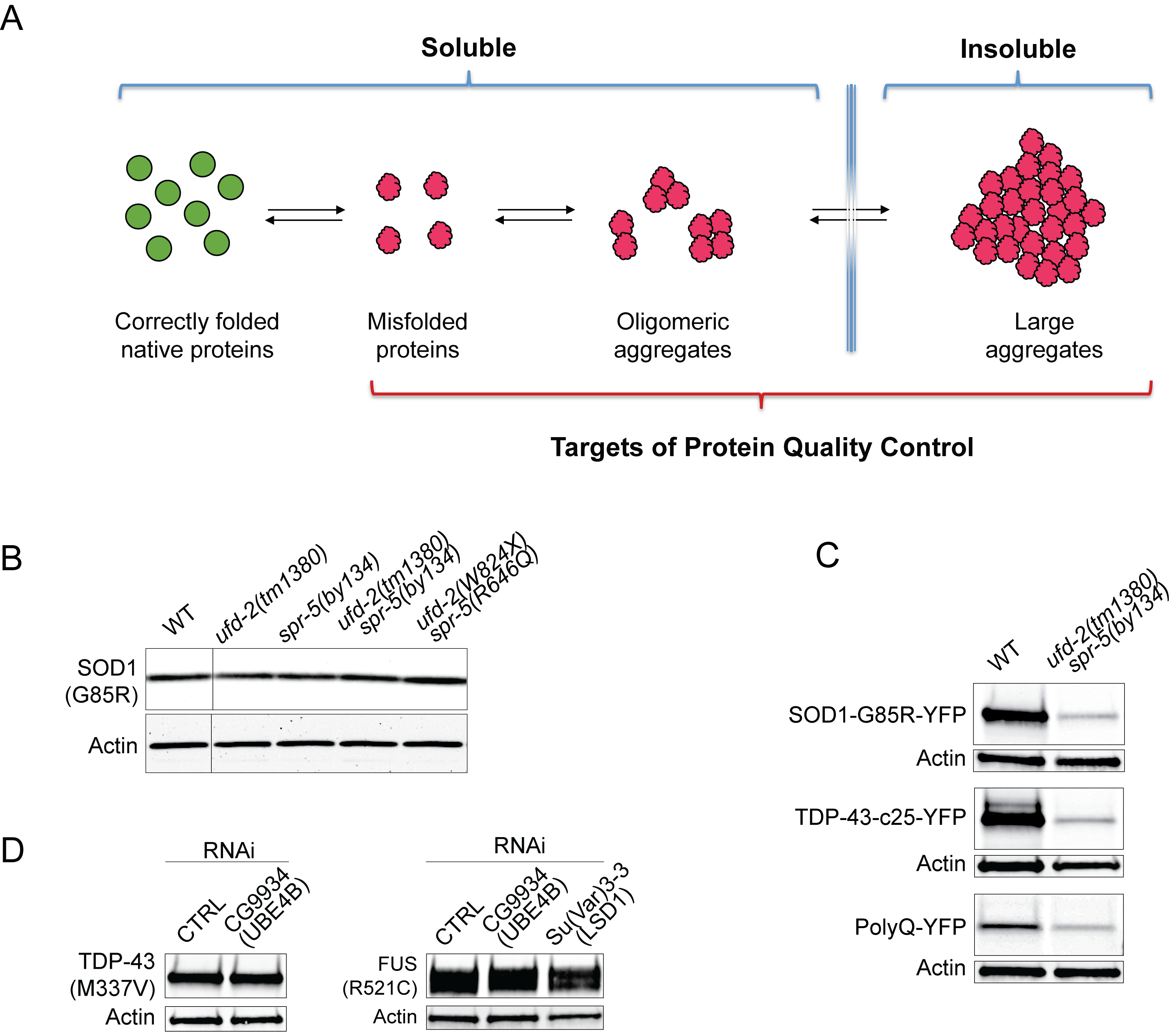

Supplement: S1 Fig — (A) A schematic for protein misfolding and aggregation in relation to protein solubility and quality control. For an aggregation-prone protein, there is a dynamic equilibrium among correctly folded native proteins, its misfolded forms, oligomeric aggregates, and large aggregates. The relative proportion of these species depends on the intrinsic folding properties of the protein and the cellular environment of protein quality control. In a protein solubility assay, large aggregates can be sedimented into the insoluble fraction via extraction and centrifugation, while smaller oligomeric aggregates and other misfolded proteins are retained in the soluble fraction. The misfolded and aggregated proteins are targets of cellular protein quality control machineries including molecular chaperones, the ubiquitin-proteasome system, and autophagy. (B) Western blotting analyses of C. elegans indicate that the ufd-2 and spr-5 mutations do not substantially alter the total protein levels of untagged SOD1G85R. The western blot lanes are from the same gel and exposure. (C) Total protein levels of YFP-tagged SOD1G85R, TDP-43c25, or PolyQ proteins are reduced in double ufd-2 and spr-5 mutant strains, suggesting that larger fractions of YFP-tagged misfolded proteins are removed in the mutant strains. (D) Total protein levels of untagged TDP-43M337V or FUSR521C are not substantially altered in Drosophila with the knockdown of ufd-2/UBE4B (CG9934) or spr-5/LSD1 (Su(Var)3-3). (TIF) [file pbio.1002114.s002.tif]

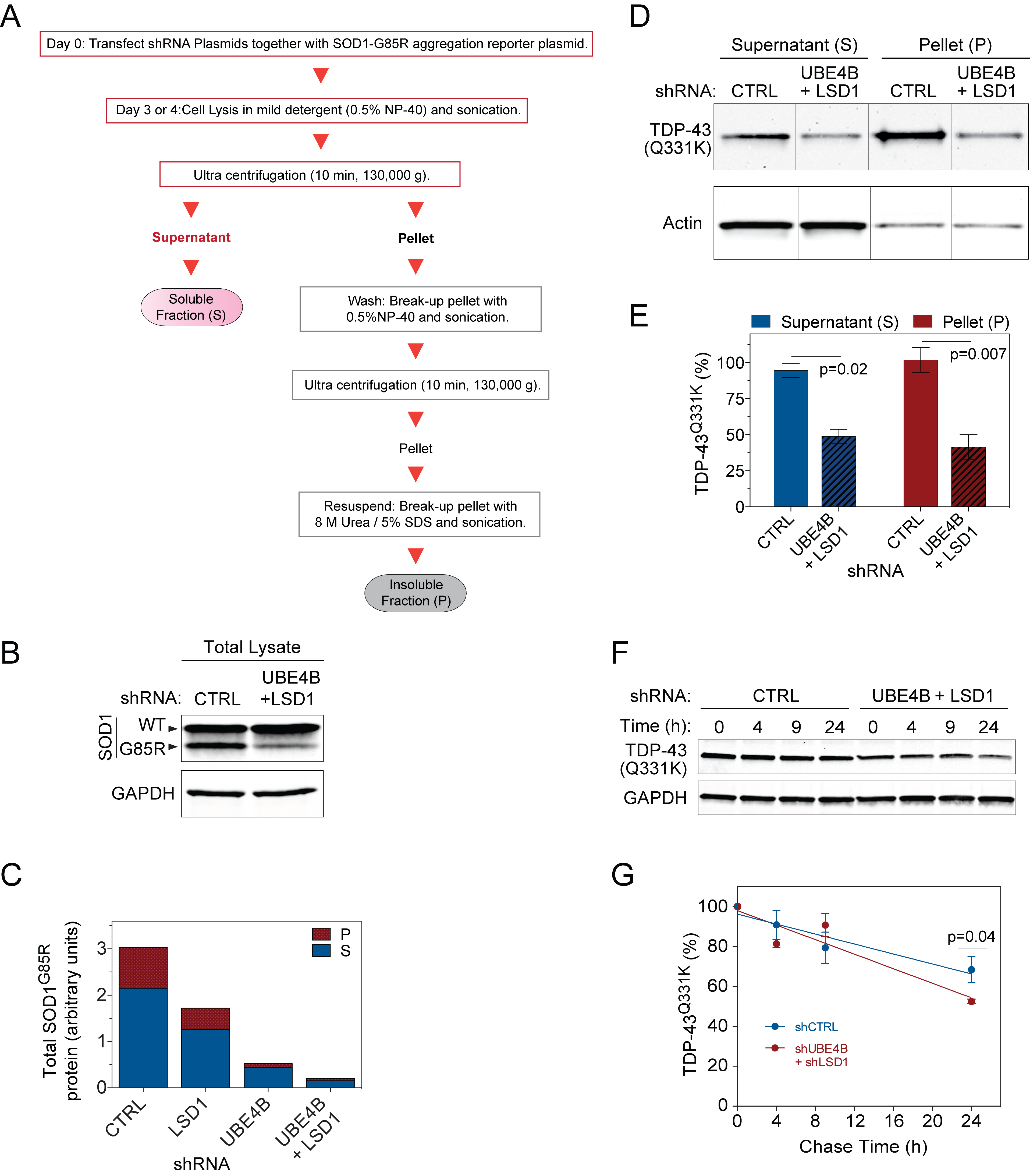

Supplement: S2 Fig — (A) The flow chart of the mammalian cell-based protein solubility assay as described in the Materials and Methods. (B) A representative western blot of total SOD1G85R protein in HEK293T cells treated with UBE4B and LSD1 shRNAs. (C) Quantification of total SOD1G85R protein and its corresponding supernatant (S) and pellet (P) fractions. The quantification is based on the band intensities and the relative amounts of S and P fractions as parts of the total lysate that were loaded on the gel. The percentage of insoluble SOD1G85R protein in the total SOD1G85R protein remains relatively stable at 25%–30%. (D) A representative western blot of the TDP-43Q331K protein solubility assay. HEK293T cells were transfected with a TDP-43Q331K expression plasmid, together with a control shRNA (CTRL) or mixed UBE4B and LSD1 shRNA plasmids. Following cell lysis and fractionation, S and P fractions were run on 15% SDS-PAGE gels. The western blot lanes are from the same gel and exposure. (E) Quantification of TDP-43Q331K in the S and P fractions, containing smaller and larger aggregates, respectively, shows a significant reduction in protein levels caused by the UBE4B and LSD1 knockdown (n = 2 for S, n = 3 for P). (F) Western blot analyses of cycloheximide chase show that TDP-43Q331K protein is degraded faster in double UBE4B and LSD1 knockdown cells than in the nontargeting shRNA control cells. (G) Quantification of chase experiments in (F) (n = 2). Data represent means ± SEM. The numerical data used to make this figure can be found in S1 Data. (TIF) [file pbio.1002114.s003.tif]

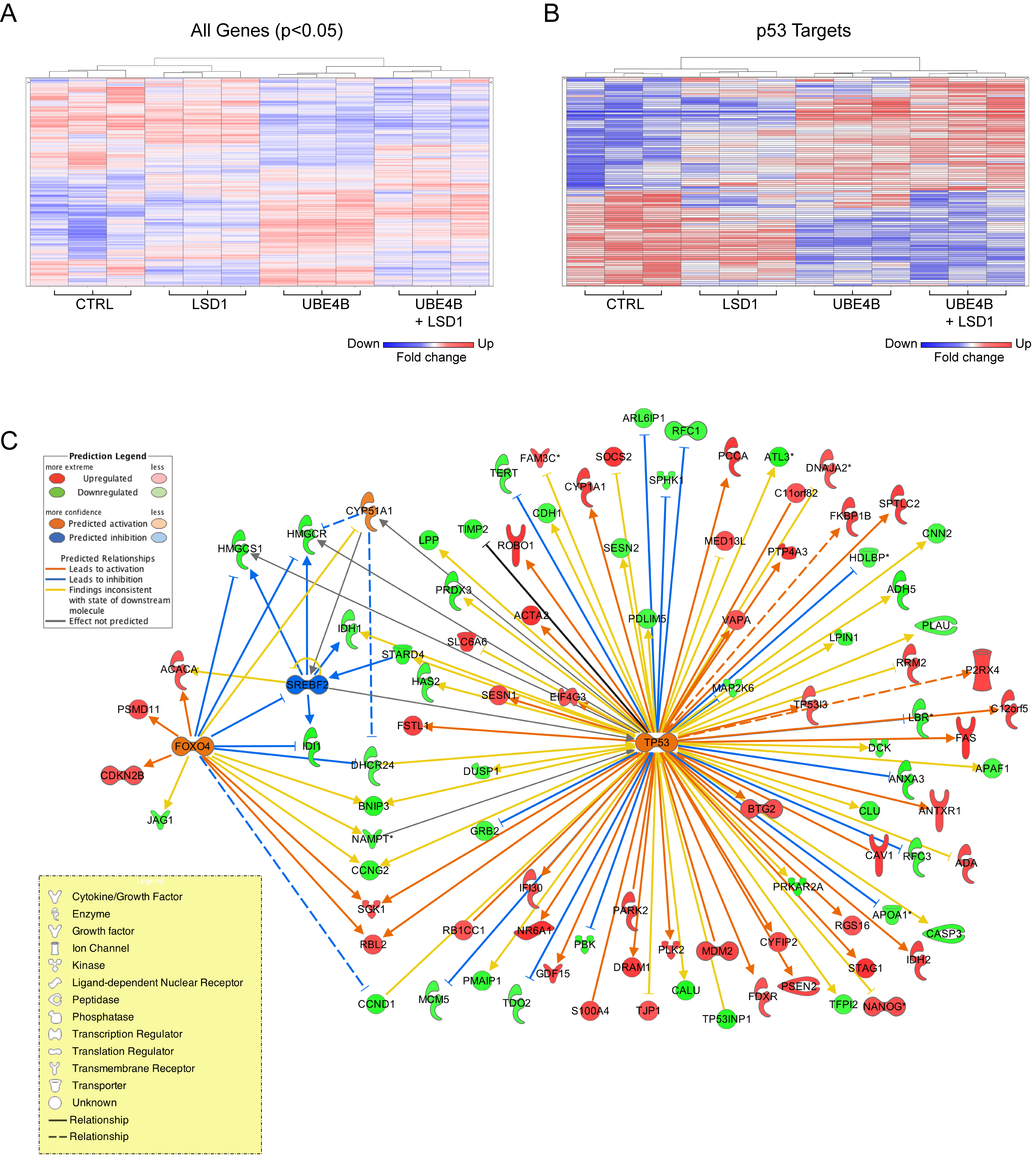

Supplement: S3 Fig — (A) The heat map of microarray signals of differentially regulated genes (p < 0.05) upon the knockdown of LSD1 alone, UBE4B alone, or both, in triplicates. Hierarchical clustering of the samples indicates that the single UBE4B knockdown induces similar transcriptional changes as the double knockdown, consistent with the pattern of antiproteotoxic activities shown in Fig. 3A and 3B. (B) The heat map of p53 transcriptional targets. The hierarchical clustering of the samples demonstrates the same pattern as shown above for all differentially regulated genes. (C) The p53 network is activated in the UBE4B and LSD1 double-knockdown cells (p53: z-score = 2.0; p-value of overlap = 2.49 x 10^-2^). The transcriptional targets with changes consistent with p53 activation are shown, with up-regulated genes in red and down-regulated genes in green. (TIF) [file pbio.1002114.s004.tif]

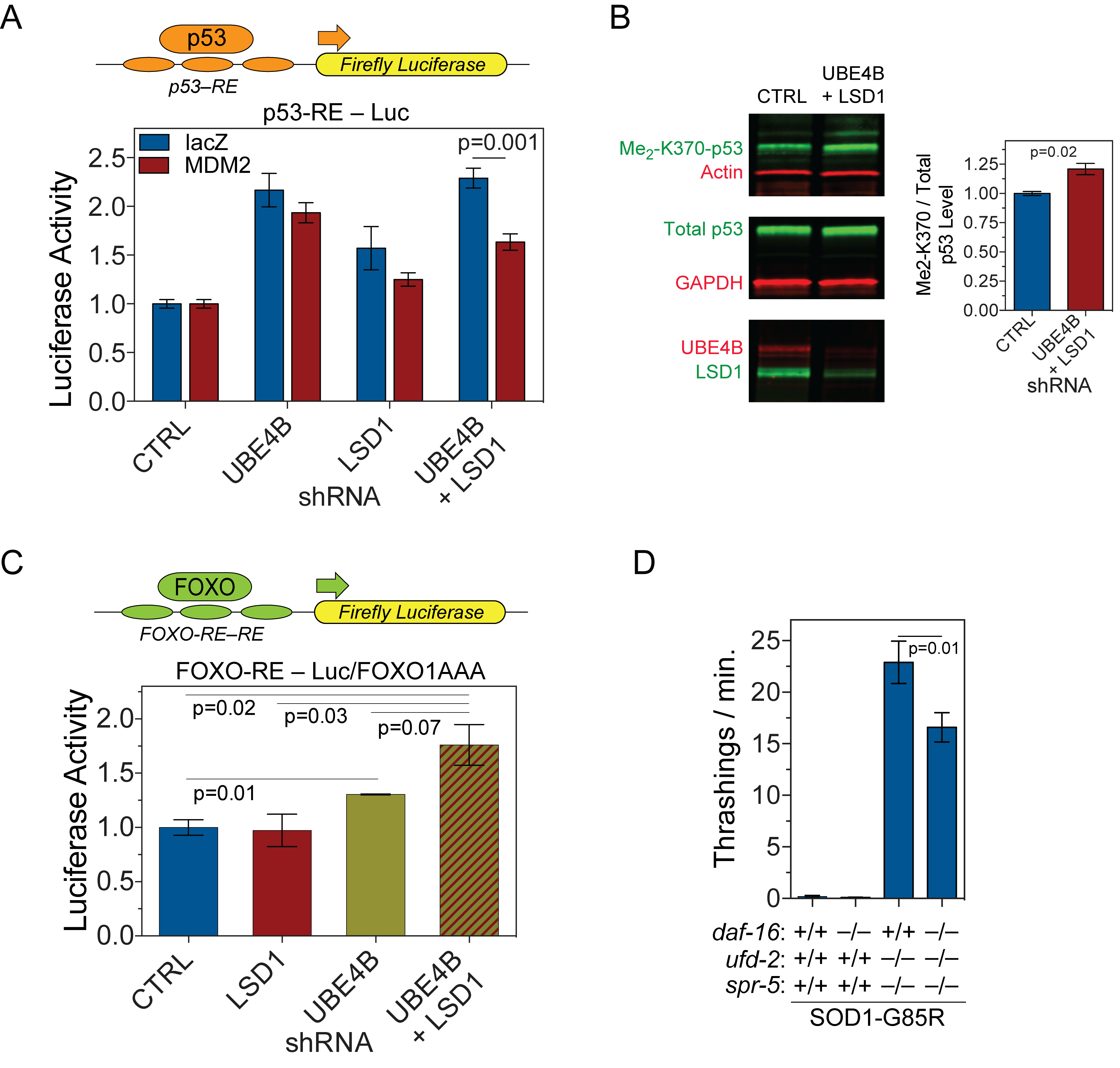

Supplement: S4 Fig — (A) Elevation of MDM2, the p53-targeting ubiquitin ligase, significantly reduced the p53 response element-mediated activity that was induced by the UBE4B and LSD1 knockdown (n = 5). MDM2 is co-transfected with the p53 reporter upon knockdown of UBE4B and/or LSD1. (B) The dimethylation at the K370 residue of the p53 protein (Me2-K370-p53) is increased in double UBE4B and LSD1 knockdown cells. In HEK293T cells treated with UBE4B and LSD1 shRNAs, there is an increase of Me2-K370-p53 relative to the total p53 protein in the nucleus-enriched fraction as shown in the western blots (left) and quantification chart (right, n = 3). (C) A constitutively active forkhead responsive element luciferase reporter (FOXO1-AAA) was used to demonstrate that the knockdown of UBE4B and LSD1 specifically induces the FOXO1 transcriptional activity (n = 3). (D) C. elegans locomotion assays indicate the rescuing effects of loss-of-function mutations spr-5(by134);ufd-2(tm1380) (indicated by-/-) on the neurotoxicity of transgenic SOD1G85R protein. Loss-of-function mutation daf-16(mu86) partially reversed locomotion rescue of spr-5(by134);ufd-2(tm1380) mutations (n > 40). Data represent means ± SEM. The numerical data used to make this figure can be found in S1 Data. (TIF) [file pbio.1002114.s005.tif]

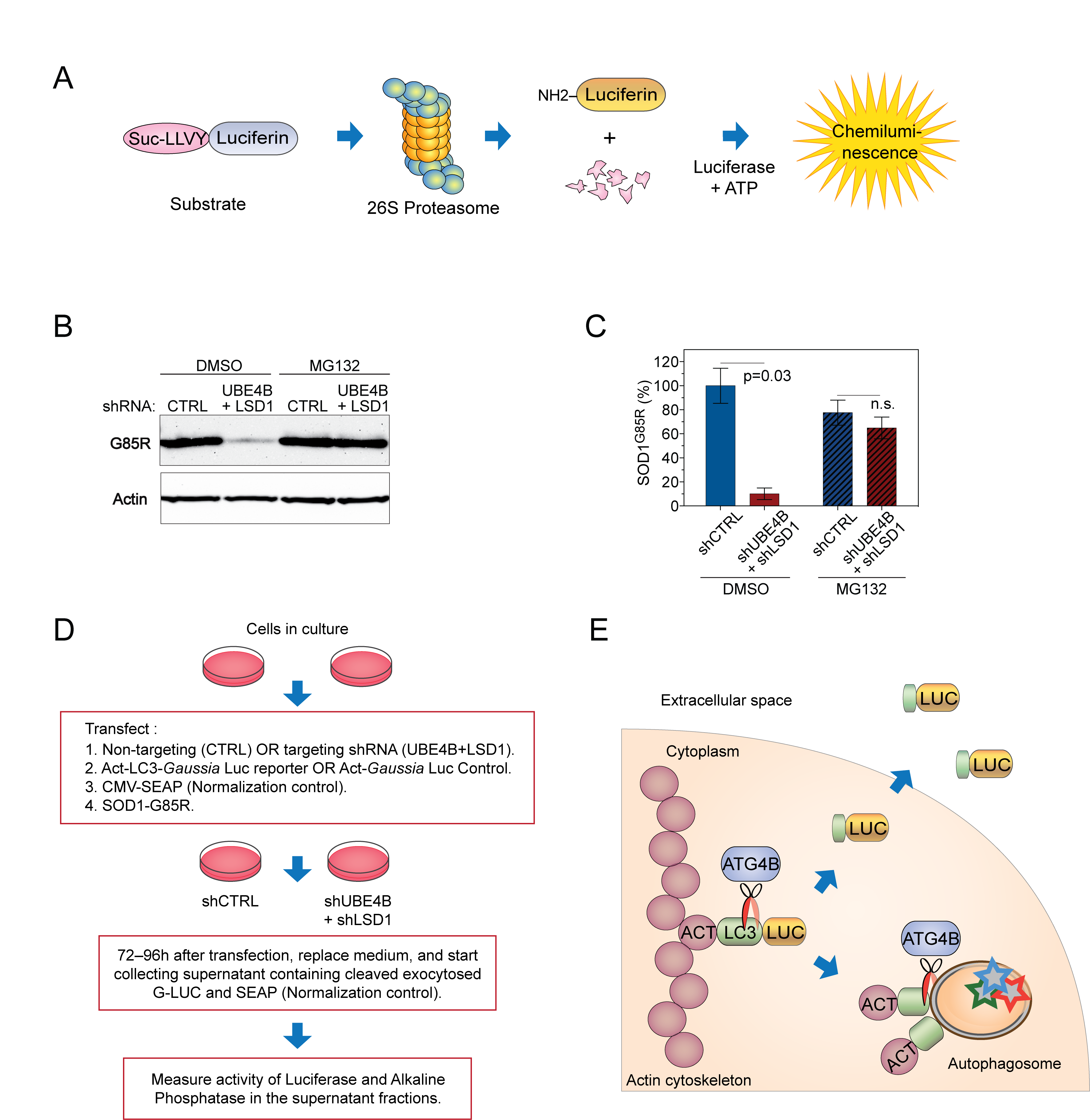

Supplement: S5 Fig — (A) A schematic of a luciferase-based proteasomal activity assay. Isolated cytosol is mixed with a peptide substrate, Suc-LLVY-luciferin. The chymotrypsin-like activity of proteasomes cleaves off the Suc-LLVY peptide, releasing the amino-luciferin, which produces strong chemiluminescence in the presence of luciferase and ATP. The detected chemiluminescence is used to quantify the proteasomal activity. (B) Inhibition of the proteasomal activity reverses the reduction of insoluble SOD1G85R protein in the pellet fraction induced by the UBE4B and LSD1 knockdown. Western blots are shown for insoluble SOD1G85R proteins from MG132-treated (20 μM) or untreated (DMSO) HEK293T cells with knockdown of UBE4B and LSD1 or nontargeting shRNA controls. Cells were treated with MG132 for 48 h and lysed 72 h post-transfection. (C) Quantification of SOD1G85R levels from western blots as shown in (B), n = 2. Data represent means ± SEM. (D) The flow chart of an autophagic activity assay to measure LC3 cleavage based on a luciferase (GLuc) reporter. Cells were transfected with a set of plasmids to knockdown LSD1 and UBE4B (or nontargeting shRNA, CTRL) and to express the GLuc reporters and SEAP (secreted embryonic alkaline phosphatase). SEAP is constitutively secreted and serves as a transfection normalization control. SOD1G85R is expressed concurrently to match the condition with the increased burden of misfolded proteins, as described earlier (Fig. 3). (E) A schematic of the LC3 cleavage and Gluc release assay. A cleavable fusion protein, Actin(Act)-LC3-GLuc, or its uncleavable negative control, Act-GLuc, is anchored to the actin cytoskeleton inside the cell. When Act-LC3-GLuc is cleaved by the autophagy-associated protease ATG4B, the GLuc fragment is released from its actin anchor and rapidly secreted out of the cell. The activity of GLuc in the cell medium is assayed over a period of several days using the Dual Luminescence Assay kit. The numerical data used to make this figure can be [file pbio.1002114.s006.tif]

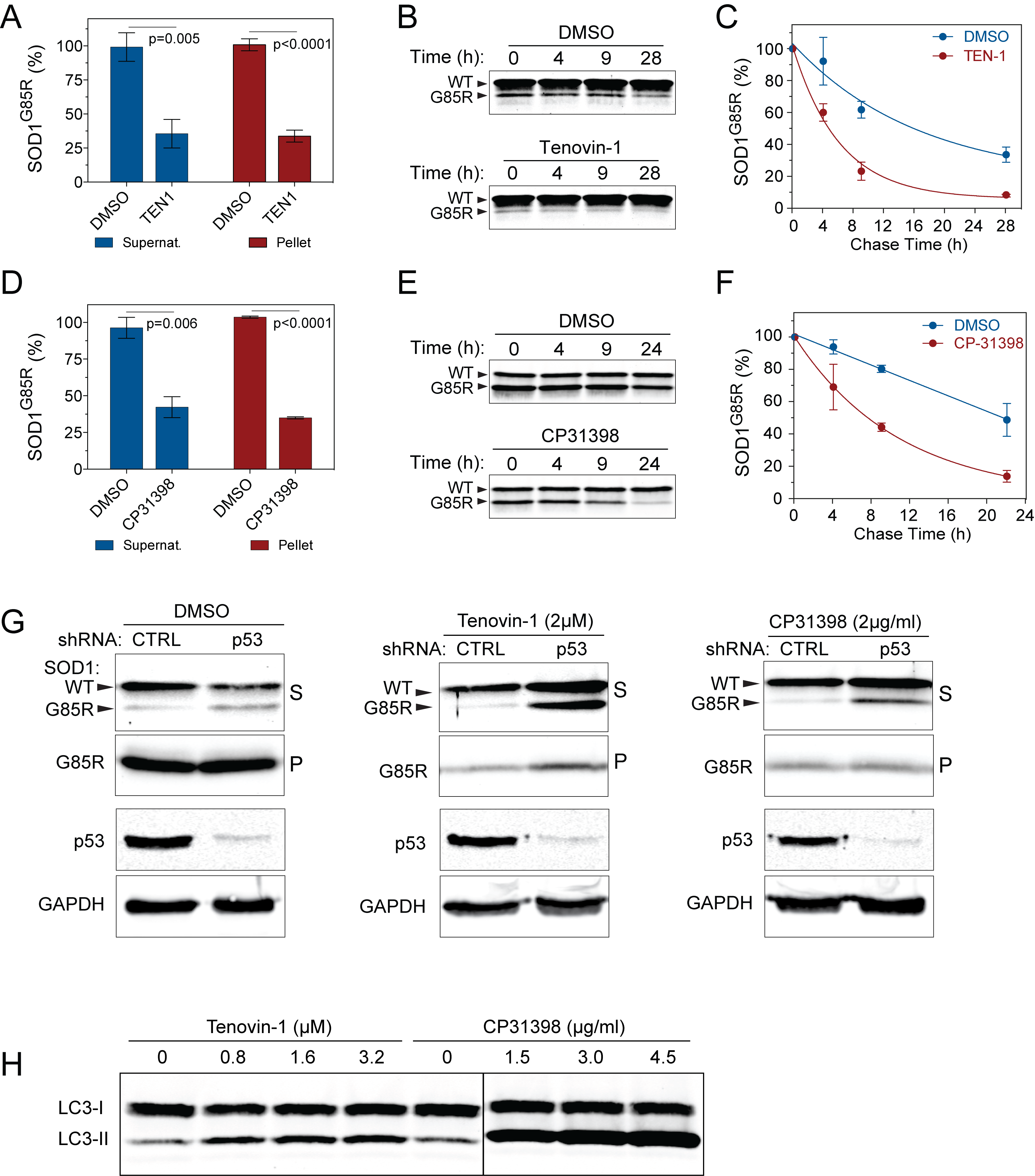

Supplement: S6 Fig — (A) The p53 small molecule activator, Tenovin-1 (TEN1, 2 μM), significantly decreased the levels of misfolded SOD1G85R but not WT SOD1 proteins in both supernatant and pellet fractions as compared with vehicle-treated controls (VEH) (n = 4). Representative western blots are shown in Fig. 6A. The SOD1G85R solubility assay in HEK293T cells is described in Materials and Methods. (B) Western blots of cycloheximide chase experiments with Tenovin-1-treated or untreated HEK293T cells. (C) Quantification of the chase experiments shows increased SOD1G85R degradation in Tenovin-1-treated cells (n = 2). (D) Another p53 small molecule activator, CP-31398 (4 μg/ml), also significantly decreased the levels of misfolded SOD1G85R proteins but not WT SOD1 proteins in both supernatant and pellet fractions (n = 3). Representative western blots are shown in Fig. 6A. (E) Western blots of cycloheximide chase experiments with CP31398-treated or untreated HEK293T cells. (F) Quantification of the chase experiments shows increased SOD1G85R degradation in CP31398-treated cells (n = 2). Data represent means ± SEM. (G) Knockdown of p53 blocked the improved clearance of misfolded SOD1G85R proteins by Tenovin-1 or CP-31398. HEK393T cells were transfected with SOD1G85R and treated with p53-activating drugs Tenovin-1 or CP31398. Protein aggregation assays were performed to evaluate the levels of SOD1G85R proteins in the supernatant (S) and pellet (P) fractions. The reduction of SOD1G85R aggregation in cells treated with Tenovin-1 or CP-31398 is dependent on p53, as the knockdown of p53 abolishes the ability of the drugs to remove aggregates. (H) p53-activating drugs activate autophagy as indicated by LC3 protein levels. HEK293T cells treated with Tenovin-1 or CP-31398 for 24 h were lysed in 1% SDS buffer, and LC3-I and LC-II levels were analyzed by western blots. For both Tenovin-1 and CP-31398, the LC-II levels are augmented with increasing drug concentrations. Both panels were from the same gel a [file pbio.1002114.s007.tif]

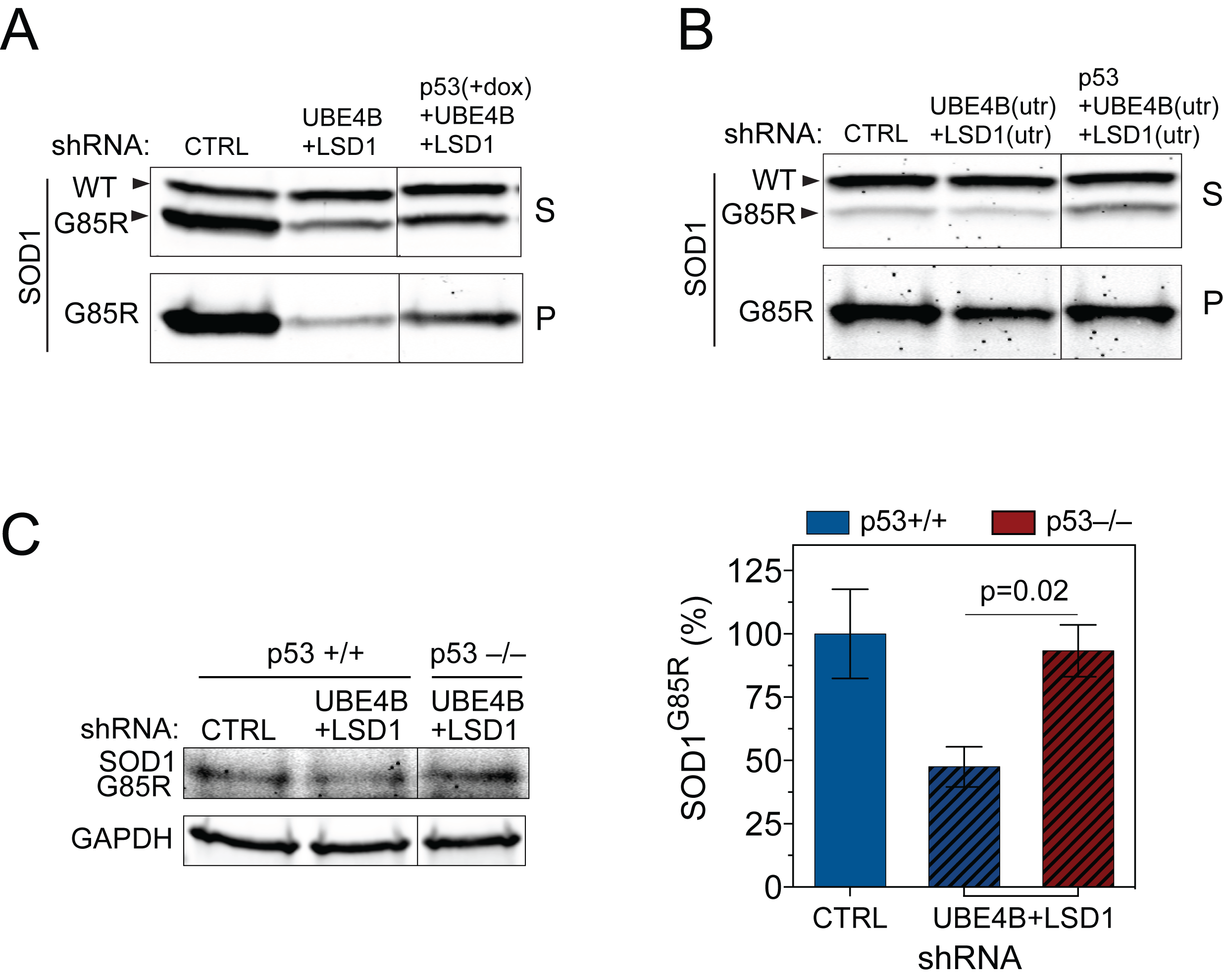

Supplement: S7 Fig — (A) Stable knockdown of p53 partially blocks the improved clearance of SOD1G85R proteins conferred by the knockdown of UBE4B and LSD1. A stable cell line with inducible knockdown of p53 via an integrated shRNA (in a pR4R3-TET-PURO vector) is used to conditionally remove p53 upon the induction of doxycycline (DOX). The protein solubility assay was used to analyze the SOD1G85R protein levels in supernatant (S) and pellet (P) fractions. The knockdown of UBE4B and LSD1 substantially enhances the clearance of misfolded SOD1G85R proteins, but this effect is partially reversed by the DOX-induced knockdown of p53 (+DOX). The western blots for the S and P fractions are from the same gels. (B) Transient knockdown of p53 blocks the improved clearance of SOD1G85R proteins conferred by the knockdown of UBE4B and LSD1. The protein solubility assay was performed as in (A), except with the knockdown of p53 achieved through shRNA transient transfection and with LSD1 and UBE4B shRNAs in a pR4R3-NEO vector targeting their respective 3′ UTRs (see Materials and Methods). (C) Loss of p53 blocks increased SOD1G85R protein clearance conferred by the knockdown of UBE4B and LSD1 in HCT116 cells. HCT116 cells with either the p53 knockout or WT genotype were treated with the UBE4B and LSD1 shRNAs or nontargeting controls. Left: western blot analyses of the insoluble SOD1G85R proteins show that loss of p53 increases the levels of the mutant SOD1 proteins, and GAPDH is a loading control. Right: quantification of the western blots indicates that p53 is required for the effect of UBE4B and LSD1 on SOD1G85R protein clearance (n = 3). Data represent means ± SEM. The numerical data used to make this figure can be found in S1 Data. (TIF) [file pbio.1002114.s008.tif]
