# Supplementary material for: Regulation of Protein Quality Control by UBE4B and LSD1 through p53-Mediated Transcription
Source: PLoS Biol. 2015 Apr 2;13(4):e1002114. doi: 10.1371/journal.pbio.1002114 (PMC4383508; doi:10.1371/journal.pbio.1002114)
Supplement: S1 Text — (DOC) [file pbio.1002114.s013.doc]

**Supporting Information**

**Materials and Methods**

*C. elegans* strains

| Strain/Allele | Genotype | Description | Reference |
| --- | --- | --- | --- |
| *iwls25* | *Psnb-1::SOD1-WT* | Human SOD1 wild-type cDNA driven by the pan-neuronal *snb-*1 promoter | [1] |
| *iwls10* | *Psnb-1::SOD1-G85R* | Human SOD1 G85R cDNA driven by the pan-neuronal *snb-1* promoter | [1] |
| *iwIs37* | *Psnb-1::SPR-5(WT)* | *C. elegans spr-5* cDNA driven by the pan-neuronal *snb-1* promoter | present study |
| *iwIs83* | *Psnb-1::UFD-2(WT)* | *C. elegans ufd-2* cDNA driven by the pan-neurona*l snb-1* promoter | present study |
| *spr-5(iw11)* | R646Q | M1 suppressor mutation | present study |
| *ufd-2(iw10)* | W824X | M1 suppressor mutation | present study |
| BR3417  *spr-5(by134)* | Y283X | non-sense mutation | [2] |
| *ufd-2(tm1380)* | ∆798 C-ter. amino acids | deletion | S. Mitani, NBRP |
| *iwls8* | *Psnb-1::SOD1-G85R-YFP* | Human SOD1-G85R-YFP fusion driven by the pan-neuronal *snb-1* promoter | [1] |
| *iwIs22* | *Psnb-1::TDP-C25-YFP* | Human TDP-43 C-terminal fragment YFP fusion driven by the *snb-1* promoter | [3] |
| AM716  (*rmIs284*) | P*rgef-1::Q67-YFP* | Polyglutamine Q67 YFP fusion driven by the neuron-specific *rgef-1* promoter | Gift from R. Morimoto |
| CF1038  *daf-16(mu86)* | Deletion allele of *daf-16* | A null allele of *daf-16* | [4] |
| CB4856 | wild-type | Hawaii strain | CGC |

Some strains were provided by the *Caenorhabditis* Genetics Center (CGC), which is funded by NIH Office of Research Infrastructure Programs (P40 OD010440). NBRP: National Bioresource Project (Japan).

*Drosophila* Strains and Assays

Flies were reared on standard yeast-agar-cornmeal medium and crosses were performed at 25°C. *Drosophila* transgenic strains carrying GAL4-inducible human ALS disease-causing alleles of FUS/TLS and TDP-43 were previously described [5,6]. Standard genetic procedures were used to generate the GMR-GAL4/CyO, tub-GAL80; UAS-FUS-hR521C/TM6B, Tb and GMR-GAL4, UAS-hTDP-43-M337V/CyO, tub-GAL80 transgenic strains. The following *Drosophila* strains were obtained from the Bloomington Stock Center: GAL4-inducible RNAi knockdown of CG9934 (y^1^ v^1^; P{y^+t7.7^ v^+t1.8^=TRiP.JF02691}attP2), Su(Var)3-3 (y^1^ sc* v^1^; P{y^+t7.7^ v^+t1.8^=TRiP.HMS00638}attP2), or Dmp53 (y^1^ v^1^; P{y^+t7.7^ v^+t1.8^=TRiP.GL01220}attP40), which are the *Drosophila* orthologs of the human UBE4B, LSD1, or p53, respectively; a GAL4-inducible and dominant negative (DN) form of Dmp53, p53.R155H (y^1^ w^1118^; P{w^+mC^=UAS-p53.R155H.Ex}2/T(2;3)TSTL, CyO: TM6B, Tb^+^) [7]. The dominant effects of the reduction of CG9934, Su(Var)3-3, or Dmp53, as well as the induction of DN p53.R155H, on the degenerative eye phenotypes of GMR-GAL4;UAS-FUS-hR521C and GMR-GAL4;UAS-hTDP-43-M337V strains were assessed two weeks after the crosses were performed. Qualitative changes in ommatidial structure and glossiness phenotypes were monitored for enhancement or suppression. For *Drosophila* total protein lysates an equal number of fly heads were homogenized with pestle in buffer (8 M Urea, 5% SDS, 40 mM Tris-Cl pH 6.8, 0.1 mM EDTA, and proteinase inhibitors [Sigma, P8340]), followed by 5 min sonication in Diagenode Bioruptor (High, 30-sec pulse, 30-sec pause). Lysates were cleared by 10 min 21,000 *g* centrifugation and transferred to fresh tubes. For the eye pigmentation assay, the relative pigment amounts were determined spectrophotometrically with absorption at 480 nm (pigment) and 600 nm (background) using total fly head protein lysates.

Suppressor Mapping and Mutation Identification

The suppressor mutations were assigned to chromosomal locations through linkage mapping using single nucleotide polymorphisms between the wild-type strains N2 Bristol and CB4856 Hawaii. The unique mutations within the mapping intervals were identified by deep-sequencing and comparing the genomes of the M1 suppressor mutant and the parental strain carrying the SOD1 transgene. The genome sequencing data was analyzed with a bioinformatic pipeline containing Bowtie 2 [8], SAMtools [9], SnpEff [10], and the Integrative Genomics Viewer (IGV) [11,12]. The identified mutations were confirmed by Sanger sequencing of the PCR-amplified loci. The phenotype-causing mutations were validated by independent alleles of the candidate genes.

Whole Genome Sequencing was performed by the Johns Hopkins Deep Sequencing & Microarray Core Facility. *C. elegans* genomic DNA was purified using the DNeasy Blood and Tissue Kit (Qiagen). The deep sequencing was carried out on an Illumina HiSeq platform. Bowtie 2 was used to index the *C. elegans* reference genome at University of California, Santa Cruz, and to align the Illumina sequencing reads to this reference. Using Bowtie 2, a Sequence Alignment/Map (SAM) output was obtained and used in subsequent analyses. The SAMtools software package was used to identify variants and call SNPs and INDELS based on the SAM alignment files. Variants were called and written to a VCF (Variant Call Format) file. SnpEff was then used to annotate the effects of variants on protein coding. Finally, the IGV browser was used to view the variants and the underlying sequence reads.

shRNAs

Gene knockdown in mammalian cells was achieved by transiently expressing shRNA plasmids, or stably expressing doxycycline-inducible shRNA in integrated cell lines when indicated. For the transient knockdown, we used vectors expressing an shRNA with an RFP marker (Origene, pRFP-C-RS), one with an EGFP marker (pLVTH) [13], or a vector constitutively expressing multiple shRNAs on a single vector backbone (pR4R3-NEO). For the stable knockdown, we constructed the vector pR4R3-TET-PURO and generated stable cell lines as described bellow.

To generate vectors with multiple shRNA expression modules on the same vector pR4R3-NEO or pR4R3-TET-PURO, we used a multi-fragment Gateway cloning system, which recombines multiple DONR constructs to a DEST plasmid. To make DONR constructs that can link multiple shRNA cassettes in tandem, we modified pP4-P1R, pP1-P2, and pP2R-P3 vectors (a gift from G. Seydoux) to contain different shRNA sequences (inserted by AgeI-HindIII digestion) under the H1-tet (H1/TO) promoter and thus generate L4R1-H1/TO-shRNA_1_, L1L2-H1/TO-shRNA_2_, and R2L3-H1/TO-shRNA_3_ DONR vectors. The H1/TO promoter itself was derived from pTET-LKO-puro [14].

To design DEST vector for constitutive shRNA expression in mammalian cells (pR4R3-NEO), we first amplified the R4-R3 Gateway cassette from the plasmid pCG150 (a gift from G. Seydoux) and inserted it into pcDNA3.1 vector (Invitrogen) by MfeI-BstBI. To generate doxycycline-inducible DEST vector (pR4R3-TET-PURO), we replaced *neo* gene of pR4R3-NEO with the pkg promoter-TET-Repressor-IRES-Puromycin cassette from the pTET-LKO-puro plasmid. Finally, we recombined two or three H1/TO-shRNA plasmids into the recipient Destination vector pR4R3-tet-puro to generate final tet-inducible shRNA plasmids with multiple shRNA expression modules.

Stable mammalian cell lines were generated by linearizing the pR4R3-TET-PURO shRNA plasmid, transfecting it into HEK293T cells, and selecting for puromycin-resistant colonies. Clones were further selected for effective knockdown of UBE4B, LSD1 and p53 genes upon induction with doxycycline.

For simultaneous knockdown with multiple shRNAs, the total amounts of shRNAs were adjusted equal by nontargeting control shRNAs.

**The shRNA target sequences in this study are listed in the table below.**

| Gene | shRNA plasmid | Target sequence | Source |
| --- | --- | --- | --- |
| nontargeting | shCTRL-RFP-C-RS | gcactaccagagctaactcagatagtact | Origene |
| nontargeting | shNT-ENTRY | gcgcgatagcgctaataattt | present study |
| nontargeting | shCTRL-LVTH | gcactaccagagctaactct | present study |
| LSD1 | shLSD1-RFP-C-RS #45 | agccacctgacagtaaggaatggctactc | Origene |
| LSD1 | shLSD1-RFP-C-RS #46 | gatactgtgcttgtccaccgagttcacag | Origene |
| LSD1 | shLSD1-RFP-C-RS #47 | ggatttggcaaccttaacaaggtggtgtt | Origene |
| LSD1 | shLSD1-RFP-C-RS #48 | tcaaagatgagcagattgaacattggaag | Origene |
| LSD1 | shLSD1.UTR-ENTRY | ggagctcctgatttgacaaag | present study |
| UBE4B | shUBE4B-RFP-C-RS #69 | catttcaccattgaaacctgcaaagagac | Origene |
| UBE4B | shUBE4B-RFP-C-RS #70 | agagtcttggtctcaatgtccacaacatg | Origene |
| UBE4B | shUBE4B-RFP-C-RS #71 | taacgcctttcaccattaagaggaaagcg | Origene |
| UBE4B | shUBE4B-RFP-C-RS #72 | gcagtcagccagcttctgagcaacatccg | Origene |
| UBE4B | shUBE4B.UTR-ENTRY | cccgcttcctgtacatatatt | present study |
| p53 | shp53-LVTH | cccggacgatattgaacaat | [14] |

Mammalian Cell Lines, Transfections, and Drug Treatments

HEK293T and HCT116 cell lines were grown at 37˚C / 5% CO_2_ in standard DMEM medium, supplemented with 10% FBS, 2 mM L-glutamine and 1x non-essential amino acids (DMEM/10).

Transfections of mammalian cells were performed using Lipofectamine 2000 (Invitrogen), according to the manufacturer’s recommendations. For shRNAs, transfections of HEK293T cells were performed by plating 3.2x10^5^ cells in 60 mm poly(ethyleneimine) (PEI, 10 µg/ml in PBS, Sigma)-pretreated dishes one day before the transfection. 4 µg of shRNA-encoding plasmids, 350 ng of SOD1 reporter (BOS-SOD1-G85R), and 10 µl Lipofectamine 2000 (Invitrogen) were mixed in 500 µl Opti-MEM I (Invitrogen), and applied to cells in 2.5 ml Opti-MEM I. One day post transfection, medium was replaced with DMEM/10. Cells were lysed 72–96 h after the start of transfections for analysis, or transfected with additional reporter plasmids for transcriptional, proteasomal, and autophagic activity assays.

Tenovin-1 (Tocris) was resuspended at 20 mM in DMSO, and CP-31398 (Tocris) at 15 mg/ml in water. 3-Methyladenine (3-MA, Sigma) was resuspended at 10 µM in complete DMEM, by heating to 37˚C and vigorous vortexing. The use of 3-MA with full medium growth conditions has been previously shown to activate autophagy [15,16]. All drugs were diluted in DMEM/10 prior to cell treatments.

Immunoprecipitation, Western Blots, and Antibodies

Equal amounts of proteins were electrophoresed on 15% or 4–20% Tris-Cl gels (Biorad). Proteins were transferred to nitrocellulose and immuno-probed with following antibodies: mouse anti-GFP JL-8 (Clonetech, 1:2,000), rabbit anti-SOD1-100 (Enzo, 1:3,000), rabbit-anti-LSD1 (AbCam, 1:2,000), mouse anti-UBE4B (BD Transduction Labs, 1:2,000), mouse anti-p53 DO-1 (Sigma, P6874, 1:5,000), rabbit anti-p53 7F5 (Cell Signaling Technology, #9282, 1:1,000), rabbit anti-53BP1 (Cell Signaling Technology, #4937, 1:1,000), rabbit anti-Me_2_-K370-p53 (Ameritech, 1:1,000), rabbit anti-GAPDH (Pierce, 1:5,000), mouse anti-Myc 9E10 (DSHB, Univ. of Iowa,1:2,000), mouse anti-Actin C4 (Santa Cruz Biotech. 1:5,000), rabbit anti-LC3 D11 (Cell Signaling Technology, 1:2,000), rabbit anti-20Sα3 H-125 (Santa Cruz Biotech. 1:1,000), rabbit anti-S5a/PSMD4 (Enzo, 1:1,000), mouse anti-20S β1 (Enzo, 1:2,000) rabbit anti-20S β5 (Enzo, 1:2,000), and rabbit anti-PSMD11 (Bethyl, 1:1,000). Proteins were visualized using Li-Cor anti-mouse and anti-rabbit 680 and 800 fluorescent antibodies, and visualized and quantified using Odyssey scanner and Image Studio 2.0 software (Li-Cor).

For immunoprecipitation, mock-knockdown or double-knockdown HEK293T cells were lysed for 30 min at 4˚C, in IP buffer (50mM Tris pH7.5, 150mM NaCl, 0.5% Triton-X100, 2mM MgCl_2_, 100U/ml Benzonase (Sigma, E1014), 1:100 Proteinase Inhibitors (Sigma P8340) followed by brief sonication (Bioruptor, High, 2x30 sec). Insolubles were removed by centrifugation (21,000 *g*, 30 min 4˚C), and supernatants were pre-cleared with agarose-IgG beads, for 1 h at 4˚C. About 1.7 mg of total protein was incubated (~16 h, 4˚C) with either 10 µl of rabbit anti-p53 antibody (Cell Signaling Technology, 7F5) or 10 µg of normal rabbit IgG (NeoMarkers, NC-100P). IgGs were captured using magnetic A/G beads (Pierce, #88803), washed 4x10 min with IP buffer, and eluted with boiling in 2x SDS loading buffer. Equal amount were loaded on 4–20% Tris-Glycine gel, transferred and visualized with Li-Cor’s 680RD Detection Reagent.

Protein Solubility Assay

*C. elegans* strains were collected from NGM feeding plates into M9 buffer and washed five times. Mammalian culture cells grown on 60mm plate were washed two times with cold PBS. *C. elegans* and mammalian cells were lysed in 200-300 µl of lysis buffer (50 mM Tris–HCl, pH 8.0, 1 mM ethylenediaminetetraacetic acid (EDTA),100 mM NaCl and 0.5% NP-40, 1/100th protease inhibitor cocktail (Sigma, P8340) and 25 mM iodoacetamide (Sigma, I6125), sonicated on ice in Diagenode Bioruptor (High, 30-sec pulse, 30-sec pause, 5 min total). Lysates were centrifuged 5–10 min at ~130,000 *g* (25 psi) in Airfuge (Coulter-Beckman), to separate larger pelleted aggregates (P1), from soluble proteins and smaller aggregates (S1). P1 pellet was resuspended in lysis buffer and sonicated as described above, except 10 min. After centrifugation (Airfuge, ~130,000 *g*, 5–10 min), pellet (P2) was resuspended in 100 µl Urea/SDS buffer (8 M Urea, 5% SDS, 40 mM Tris-Cl pH 6.8, 0.1 mM EDTA), followed by 5 min sonication.

Microarray Transcriptome Analysis

Total RNA was isolated from 293T cells grown on 60 mm dishes using the RNeasy Mini kit coupled with an on-column genomic DNA digestion (Qiagen). The genomic DNA was further removed using Ambion’s Turbo DNA-free kit according to the manufacturer’s instructions. Total RNA was used in labeling reactions with the 3’ IVT Express labeling kit (Affymetrix). Briefly, double stranded cDNA was synthesized using 100 ng of total RNA, and further used as template to transcriptionally label and linearly amplify cell’s total RNA complement. Amplified RNA was fragmented and hybridized to the Affymetrix's human GENE 1.0ST array chip. Fluorescent signals from the hybridized probes were detected using the Affymetrix G3000 GeneArray Scanner, and analysis was performed through the Affymetrix GeneChip Command Console version 3.4 software.

The microarray data were managed and analyzed using Partek Genomic Suite (Partek) and Spotfire DecisionSite software (TIBCO Software). For the Gene Ontology analysis, the annotation file for the human genome was downloaded from the website of the Gene Ontology Consortium August, 2011.

The network analysis and upstream regulator analysis were performed using Ingenuity Pathways Analysis Software (IPA, Ingenuity Systems). For Network and Upstream Regulators analysis, the microarray data set containing gene identifiers and expression values was uploaded into the application. Each identifier was then mapped to its corresponding gene product in the Ingenuity Knowledge Base. The molecules with expression fold changes above the threshold (≥1.2), and the p-values <0.05, were overlaid onto a global molecular network developed from information contained in the Ingenuity Knowledge Base. The relevant networks of selected molecules were then algorithmically generated based on their connectivity. Similarly, for the Upstream Regulator analysis, experimental, microarray-derived expression patterns were analyzed and compared to the literature-derived expression patterns resulting from activation/inhibition of known, upstream regulatory molecules, such as, transcriptional factors, signal transducers, receptors, or chemical effectors. The probability of significant overlap between microarray-derived and literature-derived sets was set to <0.05, unless indicated otherwise. The significant agreement between the literature-predicted versus microarray-derived activation/inhibition states of an upstream regulator, or the z-score, was set ≥2.0.

Spinal Motor Neuron Survival Assay

The pregnant rat dams were euthanized by overdosing with nembutal (Children¹s Hospital of Philadelphia IACUC approved protocol # 597). Spinal cords from embryonic day 15 rats were dissociated and grown on cortical astrocyte monolayers established from 1–3-day-old rats. The spinal cord neurons were maintained in astrocyte-conditioned medium with neurotrophic factors, including ciliary neurotrophic factor, cardiotrophin-1, brain-derived neurotrophic factor, neurotrophin 4, and glial-derived neurotrophic factor (10 ng/ml, Alomone Labs). At day 13 post-isolation, the spinal cord culture was treated with a test drug or vehicle for 24 h before infection with a neuron-specific HSV vector expressing mutant SOD1 that induces neuronal death. The medium was replaced with drug or vehicle three times per week. At day 5 post-infection, motor neuron survival was measured after cell fixation and immunostaining with a motor neuron-specific antibody against neurofilament H (NF-H), SMI-32 (Covance). The motor neurons were quantified blindly in four random fields of view for each condition.

**References**

1. Wang J, Farr GW, Hall DH, Li F, Furtak K, et al. (2009) An ALS-Linked Mutant SOD1 Produces a Locomotor Defect Associated with Aggregation and Synaptic Dysfunction When Expressed in Neurons of Caenorhabditis elegans. PLoS Genet 5: e1000350. doi:10.1371/journal.pgen.1000350.

2. Eimer S, Lakowski B, Donhauser R, Baumeister R (2002) Loss of spr-5 bypasses the requirement for the C.elegans presenilin sel-12 by derepressing hop-1. EMBO J 21: 5787–5796. doi:10.1093/emboj/cdf561.

3. Zhang T, Mullane PC, Periz G, Wang J (2011) TDP-43 neurotoxicity and protein aggregation modulated by heat shock factor and insulin/IGF-1 signaling. Hum Mol Genet 20: 1952–1965. doi:10.1093/hmg/ddr076.

4. Lin K, Dorman JB, Rodan A, Kenyon C (1997) daf-16: An HNF-3/forkhead family member that can function to double the life-span of Caenorhabditis elegans. Science 278: 1319–1322.

5. Lanson NA, Maltare A, King H, Smith R, Kim JH, et al. (2011) A Drosophila model of FUS-related neurodegeneration reveals genetic interaction between FUS and TDP-43. Hum Mol Genet 20: 2510–2523. doi:10.1093/hmg/ddr150.

6. Ritson GP, Custer SK, Freibaum BD, Guinto JB, Geffel D, et al. (2010) TDP-43 mediates degeneration in a novel Drosophila model of disease caused by mutations in VCP/p97. J Neurosci 30: 7729–7739. doi:10.1523/JNEUROSCI.5894-09.2010.

7. Ollmann M, Young LM, Di Como CJ, Karim F, Belvin M, et al. (2000) Drosophila p53 is a structural and functional homolog of the tumor suppressor p53. Cell 101: 91–101. doi:10.1016/S0092-8674(00)80626-1.

8. Langmead B, Salzberg SL (2012) Fast gapped-read alignment with Bowtie 2. Nat Meth 9: 357–359. doi:10.1038/nmeth.1923.

9. Li H, Handsaker B, Wysoker A, Fennell T, Ruan J, et al. (2009) The Sequence Alignment/Map format and SAMtools. Bioinformatics 25: 2078–2079. doi:10.1093/bioinformatics/btp352.

10. Cingolani P, Platts A, Wang LL, Coon M, Nguyen T, et al. (2012) A program for annotating and predicting the effects of single nucleotide polymorphisms, SnpEff: SNPs in the genome of Drosophila melanogaster strain w1118; iso-2; iso-3. Fly 6: 80–92. doi:10.4161/fly.19695.

11. Thorvaldsdóttir H, Robinson JT, Mesirov JP (2013) Integrative Genomics Viewer (IGV): high-performance genomics data visualization and exploration. Brief Bioinform 14: 178–192. doi:10.1093/bib/bbs017.

12. Robinson JT, Thorvaldsdóttir H, Winckler W, Guttman M, Lander ES, et al. (2011) Integrative genomics viewer. Nat Biotechnol 29: 24–26. Available: http://eutils.ncbi.nlm.nih.gov/entrez/eutils/elink.fcgi?dbfrom=pubmed&id=21221095&retmode=ref&cmd=prlinks.

13. Wiznerowicz M, Trono D (2003) Conditional suppression of cellular genes: lentivirus vector-mediated drug-inducible RNA interference. J Virol 77: 8957–8961. doi:10.1128/JVI.77.16.8957-8951.2003.

14. Wiederschain D, Susan W, Chen L, Loo A, Yang G, et al. (2009) Single-vector inducible lentiviral RNAi system for oncology target validation. Cell cycle 8: 498–504. Available: http://www.landesbioscience.com/journals/cc/article/7701/.

15. Hundeshagen P, Hamacher-Brady A, Eils R, Brady NR (2011) Concurrent detection of autolysosome formation and lysosomal degradation by flow cytometry in a high-content screen for inducers of autophagy. BMC Biol 9: 38–38. doi:10.1186/1741-7007-9-38.

16. Wu Y-T, Tan H-L, Shui G, Bauvy C, Huang Q, et al. (2010) Dual role of 3-methyladenine in modulation of autophagy via different temporal patterns of inhibition on class I and III phosphoinositide 3-kinase. J Biol Chem 285: 10850–10861. doi:10.1074/jbc.M109.080796.
